# A New Group Decision-Making Technique under Picture Fuzzy Soft Expert Information

**DOI:** 10.3390/e23091176

**Published:** 2021-09-07

**Authors:** Fairouz Tchier, Ghous Ali, Muhammad Gulzar, Dragan Pamučar, Ganesh Ghorai

**Affiliations:** 1 Department of Mathematics, King Saud University, P.O. Box 22452, Riyadh 11495, Saudi Arabia; ftchier@ksu.edu.sa; 2 Department of Mathematics, Division of Science and Technology, University of Education, Lahore 54770, Pakistan; ghous.ali@ue.edu.pk; 3 Department of Mathematics, Government College University Faisalabad, Faisalabad 38000, Pakistan; 98kohly@gmail.com; 4 Department of Logistics, Military Academy, University of Defence in Belgrade, 11000 Belgrade, Serbia; 5 Department of Applied Mathematics with Oceanology and Computer Programming, Vidyasagar University, Midnapore 721102, India; math.ganesh@mail.vidyasagar.ac.in

**Keywords:** picture fuzzy set, soft expert set, virtual reality, algorithm, group decision making

## Abstract

As an extension of intuitionistic fuzzy sets, the theory of picture fuzzy sets not only deals with the degrees of rejection and acceptance but also considers the degree of refusal during a decision-making process; therefore, by incorporating this competency of picture fuzzy sets, the goal of this study is to propose a novel hybrid model called picture fuzzy soft expert sets by combining picture fuzzy sets with soft expert sets for dealing with uncertainties in different real-world group decision-making problems. The proposed hybrid model is a more generalized form of intuitionistic fuzzy soft expert sets. Some novel desirable properties of the proposed model, namely, subset, equality, complement, union and intersection, are investigated together with their corresponding examples. Two well-known operations AND and OR are also studied for the developed model. Further, a decision-making method supporting by an algorithmic format under the proposed approach is presented. Moreover, an illustrative application is provided for its better demonstration, which is subjected to the selection of a suitable company of virtual reality devices. Finally, a comparison of the initiated method is explored with some existing models, including intuitionistic fuzzy soft expert sets.

## 1. Introduction

Multi-attribute group decision making (MAGDM) is an efficient procedure that has the ability to provide the rankings for an available finite family of objects based on multiple parameters associated with these objects. A significant problem in practical decision-making processes is how to describe a numeric value to a given alternative more accurately and efficiently. Due to the existence of fuzziness in various complex decision-making real-world problems, it was not possible to describe objects with exact values. To overcome this issue, Zadeh [1] was the first who initiated the notion of a fuzzy set (FS), which is a superset of the classical set. FS actually deals with the conception of partial truth between “absolute true” and “absolute false”. The membership function that delivers the membership values to objects from closed unit interval is very important.

The theory of FSs cannot work properly in some practical situations. For instance, when an expert gives judgment on a piece of information involving non-membership degree of an object that is obtained by considering the standard negation of the membership degree. To deal with such types of difficulties, Atanassov [2] proposed the theory of intuitionistic fuzzy sets (IFSs) as a generalization of FSs. Atanassov [2] modified the definition of FSs by adding a new component (which finds the non-membership degree). Thus, an IFS has two vague components, that is, membership grade and non-membership grade, which is an important aspect to prove its increasing applicability scope in the real world. In an IFS environment, there is a limitation on membership and non-membership grades, i.e., the sum of both membership and non-membership grades is bounded by one. In the last two decades, a number of scholars have paid attention to the above-mentioned theories and have been contributed with several fruitful results in different domains, including clustering analysis, medical diagnosis and decision making [3,4,5,6].

Although, the above-mentioned theories have been effectively utilized in different domains of the real world, there exist some situations that cannot be completely described by IFSs. For example, in a voting scenario, human opinions containing more than two answers, which are: yes, no, abstain, refusal; in this situation, IFSs fail to demonstrate it accurately. In addition, an expert may give their opinion about a certain object of a universal set with respect to a certain property, such as as: 0.5 is the membership value of the object regarding given property, 0.2 is non-membership value of the object and 0.3 is the value about which he/she shows neutral behavior (means not sure about this value). This problem cannot be tackled with the existing FSs or IFSs; therefore, for dealing with such situations, Cuong [7] proposed a new idea of picture fuzzy sets (PFSs), which is a direct generalization of FS and IFS by incorporating the idea of positive, negative and neutral membership degrees of an alternative. A PFS involves membership, neutral and non-membership values, making it better to demonstrate uncertain information as compared to FSs and IFSs. To date, several studies have been presented to solve different daily-life problems under the useful framework of PFSs. For example, Singh [8] developed a correlation coefficient technique for the PFSs. Son [9] introduced a generalized distance measure for PFSs and solved the clustering problem by applying it. Some picture fuzzy aggregation operators using *t*-norm and *t*-conorm were established by Garg [10] along with their applications in multi-criteria decision making (MCDM). Afterwards, Wei [11] introduced the TODIM method for MCDM under PFS environment. Ashraf et al. [12] developed certain MAGDM approaches and studied their applications to group decision making. With a different perspective, Sahu et al. [13] utilized a hybridized distance measure based on the theories of PFSs and rough sets for the career selection of students. For more important terminologies related to PFSs, the readers may refer to [14,15,16,17,18,19,20,21,22,23].

After the production of fuzzy sets, another useful tool to handle imprecise information, called the rough set model, was developed by Pawlak [24], in 1982. Both the fuzzy set theory and rough set theory have the limitation that they are unable to handle parameterized values, thus are not effective in situations considering opinions based on different parameters. To fill this gap, Molodtsov [25] initiated the concept of soft sets (SSs). The SS model consists of the parameterized families of a universal set and acts as a strong parameterization tool when dealing with uncertainties. The work was further extended by Ali et al. [26] as an introduction of some new properties and notions of SSs. Maji et al. [27] presented a decision-making application of SSs.

With the increase in complexity of uncertain situations, more efficient models are needed to combine the strengths and capabilities of existing models. Hybrid models as combinations of existing models serve the purpose efficiently. In previous years, numerous hybrid models have been developed to deal with uncertain MAGDM situations [28,29]. For instance, by the combination of SSs and PFSs, Yang et al. [30] proposed picture fuzzy SSs (PFSSs) together with their decision-making applications. Akram et al. [31] discussed *N*-soft sets regarding hesitancy and explored their applications in MAGDM. Moreover, Alcantud and Giarlotta [32] presented necessary and possible hesitant fuzzy sets for group decision making. All the models discussed above are unsuitable for dealing with MAGDM problems involving multiple experts estimations because of their incapability to deal with multiple experts opinions in a single place. To overcome this limitation, Alkhazalah and Salleh [33] introduced the concept of soft expert sets (SESs), capable of dealing with multiple expert opinions in a single platform. Afterward, Alkhazalah and Salleh [34] extended the concepts of SESs by combining them with fuzzy theory, thus introducing fuzzy SESs. The powerful concept of SESs inspired many researchers to solve various group decision-making problems using the SESs, as in [35,36,37]. For instance, Broumi and Smarandache [38] introduced intuitionistic fuzzy SESs (IFSESs) and discussed their applications. Qudah and Hassan [39] introduced the bipolar fuzzy SES model and provided its applications. Ali and Akram [40] introduced *N*-SESs and fuzzy *N*-SESs with their applications in MAGDM situations under multinary information. Moreover, Akram et al. [41] introduced the *m*-polar fuzzy SES model by the combination of *m*-polar FSs with SESs and discussed its applications to solve MAGDM problems. Very recently, Ali et al. [42] proposed a novel hybrid model called fuzzy bipolar SESs and studied its application in group decision making.

Some of the prominent existing methods on the theory of PFSs are:

- The PFSS presented by Yang et al. [30].
- The interval-valued PFSs developed by Cuong et al. [43].
- The multi-valued PFSs proposed by Jan et al. [44].

From the above studies, it has been observed that several models, including PFSs or PFSSs or interval-valued PFSs have been proposed in the last decade to compile effectively picture fuzzy information; however, an efficient hybrid model by combining the PFSs with SESs is still unattended. The main reasons behind this construction are outlined as below:

1. The hybrid model, namely, IFSESs [38] is actually deal with two-dimensional information evaluated by multiple experts with respect to multiple parameters. This model fails to deal with the important idea of neutrality degree, which can be observed in various real-life situations when we face the experts’ opinions in different types such as yes, no, abstain, refusal. For instance, in medical diagnosis, neutrality degree can be considered, that is, specific illnesses (heart or chest problems) may not have symptoms such as headache and temperature. In a similar manner, the symptoms chest pain and stomach pain have a neutral effect on different diseases, including typhoid, malaria and viral fever.
2. The concepts of PFS and SS are combined by Yang et al. [30] to form a novel hybrid model called PFSSs but this model cannot properly deal with multiple experts. We establish a novel hybrid model called picture fuzzy SESs by combining the PFSs with SESs in order to adequately deal with multiple experts.

The major contributions of the developed picture fuzzy SES (PFSES, henceforth) model are:

1. Inspired by the strength of PFSs to deal with uncertain and vague information in real-world problems, this paper focuses on initiating a new hybrid model, namely, PFSESs, as a combination of PFSs with SESs.
2. Some of its desirable properties, namely, subset, complement, union, intersection, OR operation and AND operation are investigated via corresponding examples.
3. A decision-making algorithm is developed based on PFSESs.
4. An illustrative application is provided for the better demonstration of the proposed approach.
5. Further, to prove the efficiency and reliability, the benefits and comparison of proposed hybrid model with some existing models, including intuitionistic fuzzy SESs are explored.

For more fruitful basic notions, the readers are referred to [45,46,47,48,49,50,51].

The remaining sections of this paper are arranged as: Section 2 provides a detailed review of some fundamental notions, including SES, PFS, score function and accuracy function for PFSs and PFSSs. Section 2 presents a novel hybrid model called PFSESs as an efficient extension of PFSSs or IFSESs. Further, some basic properties and operations such as subset, complement, union, intersection, OR operation and AND operation are also investigated for PFSESs through illustrative numerical examples. Section 4 provides a daily-life application for the better demonstration of the proposed approach. Section 5 studies the benefits and comparison of presented model with existing ones, including IFSESs to prove the efficiency and reliability of our developed model. Section 6 concludes the paper and provides some future directions.

## 2. Preliminaries

This section reviews certain essential basic concepts, including SES, PFS and PFSS, which will be useful for the remaining sections of the paper.

**Definition** **1** ([33]). *Let X be a universe, Q be a set of parameters and E be a set of experts. Let OP be a set of opinions such that OP={0=disagree,1=agree}. Let S=Q×E×OP and A⊆S. A pair (η,A) is referred to as a soft expert set or SES on X, where η is given as:*

η:A→P(X)

*where P(X) represents the set of all subsets of X.*

**Definition** **2** ([7]). *Let X be a universe. A picture fuzzy set O is defined as:*

$$O=\{\langle x,\zeta_{O}\left(x\right),\varrho_{O}\left(x\right),\gamma_{O}\left(x\right)\rangle :x\in X\}$$

*where $\zeta_{O}\left(x\right)\in \left[0,1\right]$ is a positive membership grade, $\varrho_{O}\left(x\right)\in \left[0,1\right]$ is a neural membership grade and $\gamma_{O}\left(x\right)\in \left[0,1\right]$ is a negative membership grade that satisfy the property given below:*

$$0\leq \zeta_{O}\left(x\right)+\varrho_{O}\left(x\right)+\gamma_{O}\left(x\right)\leq 1$$

*Here $\tau_{O}\left(x\right)=1-\left(\zeta_{O}\left(x\right)+\varrho_{O}\left(x\right)+\gamma_{O}\left(x\right)\right)$ is known as a refusal membership function of the object x∈X. For convenience, assume that $P=(\zeta_{O}\left(x\right),\varrho_{O}\left(x\right),\gamma_{O}\left(x\right))$ be a picture fuzzy number (PFN). We represent the collection of PFSs on X as $P^{F(X)}$.*

**Definition** **3** ([7]). *Let $O_{1}$ and $O_{2}$ be two PFSs on a universe X. Then, their subset relation, equality, complement, union and intersection are defined as follows:*

1. $O_{1}\subseteq O_{2}\Leftrightarrow \zeta_{O_{1}}\left(x\right)\leq \zeta_{O_{2}}\left(x\right),\varrho_{O_{1}}\left(x\right)\leq \varrho_{O_{2}}\left(x\right)\;and\;\gamma_{O_{1}}\left(x\right)\geq \gamma_{O_{2}}\left(x\right),\;\mathrm{for}\;\mathrm{all}\;x\in X$*.*
2. $O_{1}=O_{2}\Leftrightarrow O_{1}\subseteq O_{2}\;and\;O_{2}\subseteq O_{1}$*.*
3. $O_{1}\cup O_{2}=\left\{\langle x,\max\left(\zeta_{O_{1}}\left(x\right),\zeta_{O_{2}}\left(x\right)\right),\min\left(\varrho_{O_{1}}\left(x\right),\varrho_{O_{2}}\left(x\right)\right),\min\left(\gamma_{O_{1}}\left(x\right),\gamma_{O_{2}}\left(x\right)\right)\rangle :x\in X\right\}$*.*
4. $O_{1}\cap O_{2}=\left\{\langle x,\min\left(\zeta_{O_{1}}\left(x\right),\zeta_{O_{2}}\left(x\right)\right),\min\left(\varrho_{O_{1}}\left(x\right),\varrho_{O_{2}}\left(x\right)\right),\max\left(\gamma_{O_{1}}\left(x\right),\gamma_{O_{2}}\left(x\right)\right)\rangle :x\in X\right\}$*.*
5. ${O_{1}}^{c}=\left\{\langle x,\gamma_{O_{1}}\left(x\right),\varrho_{O_{1}}\left(x\right),\zeta_{O_{1}}\left(x\right)\rangle :x\in X\right\}$*.*

**Definition** **4** ([10]). *Let X be a universal set, then for any PFN $P=(\zeta_{O}\left(x\right),\varrho_{O}\left(x\right),\gamma_{O}\left(x\right))$, its score and accuracy functions are given below:*

(1) $$S(P)=\zeta_{O}\left(x\right)-\varrho_{O}\left(x\right)-\gamma_{O}\left(x\right),$$

(2) $$H(P)=\zeta_{O}\left(x\right)+\varrho_{O}\left(x\right)+\gamma_{O}\left(x\right),$$

*∀ x∈X. Notice that for any two PFNs $P_{1}$ and $P_{2}$, we say that $P_{1}$ is less than $P_{2}$ if $S\left(P_{1}\right)<S\left(P_{2}\right)$, In case if $S\left(P_{1}\right)=S\left(P_{2}\right)$, then we use accuracy function to compute whether given PFNs are equal or not. Now if $H\left(P_{1}\right)<H\left(P_{2}\right)$, we say that $P_{1}$ is less than $P_{2}$ and if $H\left(P_{1}\right)=H\left(P_{2}\right)$ then $P_{1}=P_{2}$.*

**Definition** **5** ([30]). *Let X be a universe and Q be a set of parameters. For each A⊆Q, A pair (f,A) is said to be a picture fuzzy soft set or PFSS over X where f is a function given as below:*

$$f:A\to P^{F(X)}.$$

## 3. Picture Fuzzy Soft Expert Sets

This section provides the main notion of this study, namely, PFSESs together with some fundamental properties of the model that are explained by illustrative examples.

**Definition** **6.** *Let X be a universe, Q a universe of parameters, E a set of experts and OP={1=agree,0=disagree} be a set of their opinions. For each A⊆S with S=Q×E×OP, a pair (Y,A) is called a picture fuzzy soft expert set or PFSES where* Y *is a function given as:*

$$Y:A\to P^{F(X)}$$

*In set notation: the PFSES (Y,A) over the universal set X is given below:*

(Y,A)={〈a,Y(a)〉:a∈A}

*where*

$$Y\left(a\right)=\{\langle x,\zeta_{A}\left(x\right),\varrho_{A}\left(x\right),\gamma_{A}\left(x\right)\rangle :x\in X\}$$

*satisfying $0\leq \zeta_{A}\left(x\right)+\varrho_{A}\left(x\right)+\gamma_{A}\left(x\right)\leq 1$. Here $\zeta_{A}\left(x\right)\in \left[0,1\right]$ is a positive belongingness degree, $\varrho_{A}\left(x\right)\in \left[0,1\right]$ is a neutral degree and $\gamma_{A}\left(x\right)\in \left[0,1\right]$ is a negative belongingness degree.*

The tabular representation is a more precise and compact way to represent a PFSES (Y,A). Assume that $X=\{x_{1},x_{2},\ldots ,x_{n}\}$ is a universal set, and $Q=\{q_{1},q_{2},\ldots ,q_{m}\}$ is a universe of parameters about the elements of X. Let $E=\{e_{1},e_{2},\ldots ,e_{t}\}$ be a collection of experts and OP={0=disagree,1=agree} be their opinions. Then, a PFSES (Y,A) can also be presented by tabular arrangement as displayed in Table 1.

**Example** **1.** *Consider a company that wants to buy electric cars with the rapid development of the global electric commercial vehicle market in the world from six substitutes $X=\{x_{1}=Mini\;Electric,x_{2}=Tesla\;Model\;S,x_{3}=BMW\;i3,x_{4}=Hyundai\;Kona\;EV,x_{5}=Polestar\;2,$$x_{6}=Jaguar\;I-PACE\}$. To choose the best electric car, the organization needs to take the opinions of three specialists $E=\{e_{1},e_{2},e_{3}\}$. Let $Q=\{q_{1}=touch\;screens,q_{2}=funky\;design,q_{3}=rapid\;charging,q_{4}=millage\;capacity\}$ be a favorable set of parameters provided by the company to help experts in the evaluation process to fulfill their needs accordingly. The experts provide their judgments in the form of a PFSES (Y,A), which is given in Table 2 where A⊆S=Q×E×OP.*

*From Table 2, it can be readily seen that the information closed in the first cell (0.4,0.2,0.2) is explained well as: The expert $e_{1}$ agrees if the belongingness, neutrality and non-belongingness degrees of the electric car ‘$x_{1}$’ are 0.4, 0.2 and 0.2, respectively, with respect to parameter $q_{1}$, and so on for other cells of Table 2.*

We now introduce some basic operations for PFSESs, including subset, equality, complement, union, intersection, AND and OR with corresponding examples. We start with the subset relation between PFSESs.

**Definition** **7.** *Let (Y,A) and (Ω,B) be two PFSESs over X. Then (Y,A) is said to be picture fuzzy soft expert subset of (Ω,B) if*

*1*.
A⊆B,

*2*. *Y(a) is picture fuzzy soft expert subset of Ω(a) (symbolically, Y(a)⊆Ω(a)) where a∈A and Y(a) and Ω(a) are PFSs; therefore, $\zeta_{A}\left(x\right)\leq \zeta_{B}\left(x\right),\varrho_{A}\left(x\right)\leq \varrho_{B}\left(x\right)\;and\;\gamma_{A}\left(x\right)\geq \gamma_{B}\left(x\right),$ for all x∈X.*

*This subset relation is represented by $\left(Y,A\right)\hat{\subseteq }\left(\Omega ,B\right)$. We can say, (Ω,B) is a picture fuzzy soft expert superset of (Y,A).*

Let us see an illustrative numerical example of picture fuzzy soft expert subset relation:

**Example** **2.** *Consider Example 1 again and let (Y,A) and (Ω,B) be two PFSESs over X given by Table 3 and Table 4, respectively, where A and B are given as:*

$$\begin{array}{c} A=\{\left(q_{1},e_{2},1\right),\left(q_{2},e_{1},1\right),\left(q_{3},e_{1},1\right),\left(q_{4},e_{3},1\right),\left(q_{1},e_{3},0\right),\left(q_{2},e_{3},0\right),\left(q_{3},e_{2},0\right),\left(q_{4},e_{1},0\right)\}\\ B=\{\left(q_{1},e_{2},1\right),\left(q_{2},e_{1},1\right),\left(q_{3},e_{1},1\right),\left(q_{4},e_{3},1\right),\left(q_{1},e_{3},0\right),\left(q_{2},e_{3},0\right),\left(q_{3},e_{2},0\right),\left(q_{4},e_{1},0\right)\} \end{array}$$

*Clearly A⊆B and Y(a)⊆Ω(a) for all a∈A. Thus $\left(Y,A\right)\hat{\subseteq }\left(\Omega ,B\right)$.*

**Definition** **8.** *Let X be a universe. Then any two PFSESs (Y,A) and (Ω,B) over X are called equal ⇔(Y,A) is picture fuzzy soft expert subset of (Ω,B) and (Ω,B) is picture fuzzy soft expert subset of (Y,A), that is, (Y,A)=(Ω,B) if and only if $\left(Y,A\right)\hat{\subseteq }\left(\Omega ,B\right)$ and $\left(\Omega ,B\right)\hat{\subseteq }\left(Y,A\right)$.*

**Definition** **9.** *Let (Y,A) be a PFSES over the universal set X. Then, an agree-PFSES ${\left(Y,A\right)}_{1}$ on X is a picture fuzzy soft expert subset of (Y,A) which is given by*

$${\left(Y,A\right)}_{1}=\left\{Y\left(a\right):a\in Q\times E\times \left\{1\right\}\right\}.$$

**Example** **3.** *Reconsider the PFSES (Y,A) in Example 1. Then, its agree-PFSES ${\left(Y,A\right)}_{1}$ is displayed in Table 5 below:*

**Definition** **10.** *Let (Y,A) be a PFSES over the universal set X. Then, a disagree-PFSES ${\left(Y,A\right)}_{0}$ on X is a picture fuzzy soft expert subset of (Y,A) which is given below:*

$${\left(Y,A\right)}_{0}=\left\{Y\left(a\right):a\in Q\times E\times \left\{0\right\}\right\}.$$

**Example** **4.** *Reconsider the PFSES (Y,A) in Example 1. Then, its disagree-PFSES ${\left(Y,A\right)}_{0}$ is provided by Table 6.*

**Definition** **11.** *Let (Y,A) be a PFSES on the universe X. Then, complement of PFSES (Y,A) is represented by ${\left(Y,A\right)}^{c}$ and is given as ${\left(Y,A\right)}^{c}=\{\langle \left(a,Y^{c}\left(a\right)\right):a\in A\},$ where*

$$Y^{c}\left(a\right)={\left(Y\left(a\right)\right)}^{c}=\left\{\langle x,\gamma_{A}\left(x\right),\varrho_{A}\left(x\right),\zeta_{A}\left(x\right)\rangle :x\in X\right\}.$$

**Example** **5.** *Reconsider the PFSES (Y,A) as defined in Example 1. Then, its complement ${\left(Y,A\right)}^{c}$ is computed in Table 7.*

**Proposition** **1.** *Let (Y,A) be a PFSES on X. Then ${\left({\left(Y,A\right)}^{c}\right)}^{c}=\left(Y,A\right)$.*

**Proof**. Its proof is straightforward by Definition 11.   □

**Definition** **12.** *For any two PFSESs (Y,A) and (Ω,B) on X, their union, denoted by (Y,A)⋓(Ω,B), is a PFSES (G,H)=(Y,A)⋓(Ω,B) with H=A∪B and* ∀*φ∈H,*

$$G\left(\varphi \right)=\left\{\begin{array}{cc} Y(\varphi ), & \mathrm{if}\;\;\varphi \in A-B,\\ \Omega (\varphi ), & \mathrm{if}\;\;\varphi \in B-A,\\ Y(\varphi )\cup \Omega (\varphi ) & \mathrm{if}\;\;\varphi \in A\cap B, \end{array}\right.$$

*where*

$$Y\left(\varphi \right)\cup \Omega \left(\varphi \right)=\{\langle x,\max\left(\zeta_{A}\left(x\right),\zeta_{B}\left(x\right)\right),\min\left(\varrho_{A}\left(x\right),\varrho_{B}\left(x\right)\right),\min\left(\gamma_{A}\left(x\right),\gamma_{B}\left(x\right)\right)\rangle :x\in X\}.$$

**Example** **6.** *Reconsider the PFSES (Y,A) in Example 1 and let (Ω,B) be another PFSES over X with the same information as considered in Example 1, which is displayed in Table 8, where*

$$\begin{array}{c} B=\{\left(q_{1},e_{1},1\right),\left(q_{1},e_{2},1\right),\left(q_{1},e_{3},1\right),\left(q_{2},e_{1},1\right),\left(q_{2},e_{2},1\right),\left(q_{2},e_{3},1\right),\left(q_{3},e_{1},1\right),\left(q_{3},e_{2},1\right),\\ \left(q_{3},e_{3},1\right),\left(q_{4},e_{1},1\right),\left(q_{4},e_{2},1\right),\left(q_{4},e_{3},1\right),\left(q_{1},e_{1},0\right),\left(q_{1},e_{2},0\right),\left(q_{1},e_{3},0\right),\left(q_{2},e_{1},0\right),\\ \left(q_{2},e_{2},0\right),\left(q_{2},e_{3},0\right),\left(q_{3},e_{1},0\right),\left(q_{3},e_{2},0\right),\left(q_{3},e_{3},0\right),\left(q_{4},e_{1},0\right),\left(q_{4},e_{2},0\right),\left(q_{4},e_{3},0\right)\} \end{array}$$

*The union (G,H)=(Y,A)⋓(Ω,B) of PFSESs (Y,A) and (Ω,B) in tabular arrangement is given in Table 9 below.*

**Proposition** **2.** *Let $\left(Y_{1},A\right)$, $\left(Y_{2},B\right)$ and $\left(Y_{3},C\right)$ be PFSESs over X. Then*

*1*.
$\left(Y_{1},A\right)⋓\left(Y_{2},B\right)=\left(Y_{2},B\right)⋓\left(Y_{1},A\right)$ *,*
*2*.
$\left(Y_{1},A\right)⋓\left(\left(Y_{2},B\right)⋓\left(Y_{3},C\right)\right)=\left(\left(Y_{1},A\right)⋓\left(Y_{2},B\right)\right)⋓\left(Y_{3},C\right)$ *.*

**Proof.** 

1. From Definition 12, $\left(Y_{1},A\right)⋓\left(Y_{2},B\right)=\left(G_{1},H_{1}\right)$ with $H_{1}=A\cup B$ and for all $\varphi \in H_{1}$,
  $$G_{1}\left(\varphi \right)=\left\{\begin{array}{cc} Y_{1}\left(\varphi \right), & \mathrm{if}\;\;\varphi \in A-B,\\ Y_{2}\left(\varphi \right), & \mathrm{if}\;\;\varphi \in B-A,\\ Y_{1}\left(\varphi \right)\cup Y_{2}\left(\varphi \right), & \mathrm{if}\;\;\varphi \in A\cap B, \end{array}\right.$$
  where $Y_{1}\left(\varphi \right)\cup Y_{2}\left(\varphi \right)=(x,\max\left(\zeta_{A}\left(x\right),\zeta_{B}\left(x\right)\right),\min\left(\varrho_{A}\left(x\right),\varrho_{B}\left(x\right)\right),\min(\gamma_{A}\left(x\right),$$\gamma_{B}\left(x\right))),$ for all $\left(x,\zeta_{A}\left(x\right),\varrho_{A}\left(x\right),\gamma_{A}\left(x\right)\right)\in Y_{1}\left(\varphi \right),\;\left(x,\zeta_{B}\left(x\right),\varrho_{B}\left(x\right),\gamma_{B}\left(x\right)\right)\in Y_{2}\left(\varphi \right)$ satisfying $0\leq \zeta_{A}\left(x\right)+\varrho_{A}\left(x\right)+\gamma_{A}\left(x\right)\leq 1$ and $0\leq \zeta_{B}\left(x\right)+\varrho_{B}\left(x\right)+\gamma_{B}\left(x\right)\leq 1$. Similarly, by Definition 12, $\left(Y_{2},B\right)⋓\left(Y_{1},A\right)=\left(G_{2},H_{2}\right)$ with $H_{2}=B\cup A$ and for all $\varphi \in H_{2}$,
  $$\begin{array}{c} G_{2}\left(\varphi \right)=\left\{\begin{array}{cc} Y_{2}\left(\varphi \right), & \mathrm{if}\;\;\varphi \in B-A,\\ Y_{1}\left(\varphi \right), & \mathrm{if}\;\;\varphi \in A-B,\\ Y_{2}\left(\varphi \right)\cup Y_{1}\left(\varphi \right), & \mathrm{if}\;\;\varphi \in B\cap A, \end{array}\right.\\ =G_{1}\left(\varphi \right) \end{array}$$
  where $Y_{2}\left(\varphi \right)\cup Y_{1}\left(\varphi \right)=(x,\max\left(\zeta_{B}\left(x\right),\zeta_{A}\left(x\right)\right),\min\left(\varrho_{B}\left(x\right),\varrho_{A}\left(x\right)\right),\min(\gamma_{B}\left(x\right),$$\gamma_{A}\left(x\right))),$ for all $\left(x,\zeta_{B}\left(x\right),\varrho_{B}\left(x\right),\gamma_{B}\left(x\right)\right)\in Y_{2}\left(\varphi \right),\;\left(x,\zeta_{A}\left(x\right),\varrho_{A}\left(x\right),\gamma_{A}\left(x\right)\right)\in Y_{1}\left(\varphi \right)$ satisfying $0\leq \zeta_{B}\left(x\right)+\varrho_{B}\left(x\right)+\gamma_{B}\left(x\right)\leq 1$ and $0\leq \zeta_{A}\left(x\right)+\varrho_{A}\left(x\right)+\gamma_{A}\left(x\right)\leq 1$. Hence, $\left(Y_{1},A\right)⋓\left(Y_{2},B\right)=\left(Y_{2},B\right)⋓\left(Y_{1},A\right)$.
2. It follows directly with similar arguments used in part (1).

□

**Definition** **13.** *For any two PFSESs (Y,A) and (Ω,B) on X, their intersection, denoted by (Y,A)⋒(Ω,B), is a PFSES (I,J)=(Y,A)⋒(Ω,B) with J=A∩B and for all φ∈J,*

$$I\left(\varphi \right)=\left\{\begin{array}{cc} Y(\varphi ), & \mathrm{if}\;\;\varphi \in A-B,\\ \Omega (\varphi ), & \mathrm{if}\;\;\varphi \in B-A,\\ Y(\varphi )\cap \Omega (\varphi ) & \mathrm{if}\;\;\varphi \in A\cap B, \end{array}\right.$$

*where*

$$Y\left(\varphi \right)\cap \Omega \left(\varphi \right)=\{\langle x,\min\left(\zeta_{A}\left(x\right),\zeta_{B}\left(x\right)\right),\min\left(\varrho_{A}\left(x\right),\varrho_{B}\left(x\right)\right),\max\left(\gamma_{A}\left(x\right),\gamma_{B}\left(x\right)\right)\rangle :x\in X\}.$$

**Example** **7.** *Reconsider the PFSES (Y,A) in Example 1 and let (Ω,B) be another PFSES over X, which is displayed in Table 8. Then, the intersection (Y,A)⋒(Ω,B) of PFSESs (Y,A) and (Ω,B) is provided in Table 10 below.*

**Proposition** **3.** *Let $\left(Y_{1},A\right)$, $\left(Y_{2},B\right)$ and $\left(Y_{3},C\right)$ be three PFSESs on X. Then*

*1*. $\left(Y_{1},A\right)⋒\left(Y_{2},B\right)=\left(Y_{2},B\right)⋒\left(Y_{1},A\right)$,
*2*. $\left(Y_{1},A\right)⋒\left(\left(Y_{2},B\right)⋒\left(Y_{3},C\right)\right)=\left(\left(Y_{1},A\right)⋒\left(Y_{2},B\right)\right)⋒\left(Y_{3},C\right)$.

**Proof.** 

1. From Definition 13, $\left(Y_{1},A\right)⋒\left(Y_{2},B\right)=\left(I_{1},J_{1}\right)$ with $J_{1}=A\cup B$ and for all $\varphi \in J_{1}$,
  $$I_{1}\left(x\right)=\left\{\begin{array}{cc} Y_{1}\left(\varphi \right), & \mathrm{if}\;\;\varphi \in A-B,\\ Y_{2}\left(\varphi \right), & \mathrm{if}\;\;\varphi \in B-A,\\ Y_{1}\left(\varphi \right)\cap Y_{2}\left(\varphi \right), & \mathrm{if}\;\;\varphi \in A\cap B, \end{array}\right.$$
  where $Y_{1}\left(\varphi \right)\cap Y_{2}\left(\varphi \right)=\left(x,\min\left(\zeta_{A}\left(x\right),\zeta_{B}\left(x\right)\right),\min\left(\varrho_{A}\left(x\right),\varrho_{B}\left(x\right)\right),\max\left(\gamma_{A}\left(x\right),\gamma_{B}\left(x\right)\right)\right),$ for all $\left(x,\zeta_{A}\left(x\right),\varrho_{A}\left(x\right),\gamma_{A}\left(x\right)\right)\in Y_{1}\left(\varphi \right),\;\left(x,\zeta_{B}\left(x\right),\varrho_{B}\left(x\right),\gamma_{B}\left(x\right)\right)\in Y_{2}\left(\varphi \right)$ satisfying $0\leq \zeta_{A}\left(x\right)+\varrho_{A}\left(x\right)+\gamma_{A}\left(x\right)\leq 1$ and $0\leq \zeta_{B}\left(x\right)+\varrho_{B}\left(x\right)+\gamma_{B}\left(x\right)\leq 1$. Similarly, by Definition 13, $\left(Y_{2},B\right)⋒\left(Y_{1},A\right)=\left(I_{2},J_{2}\right)$ with $J_{2}=B\cup A$ and for all $\varphi \in J_{2}$,
  $$\begin{array}{c} I_{2}\left(\varphi \right)=\left\{\begin{array}{cc} Y_{2}\left(\varphi \right), & \mathrm{if}\;\;\varphi \in B-A,\\ Y_{1}\left(\varphi \right), & \mathrm{if}\;\;\varphi \in A-B,\\ Y_{2}\left(\varphi \right)\cap Y_{1}\left(\varphi \right), & \mathrm{if}\;\;\varphi \in B\cap A, \end{array}\right.\\ =I_{1}\left(\varphi \right) \end{array}$$
  where $Y_{2}\left(\varphi \right)\cap Y_{1}\left(\varphi \right)=(x,\min\left(\zeta_{B}\left(x\right),\zeta_{A}\left(x\right)\right),\min\left(\varrho_{B}\left(x\right),\varrho_{A}\left(x\right)\right),\max(\gamma_{B}\left(x\right),$$\gamma_{A}\left(x\right))),$ for all $\left(x,\zeta_{B}\left(x\right),\varrho_{B}\left(x\right),\gamma_{B}\left(x\right)\right)\in Y_{2}\left(\varphi \right),\;\left(x,\zeta_{A}\left(x\right),\varrho_{A}\left(x\right),\gamma_{A}\left(x\right)\right)\in Y_{1}\left(\varphi \right)$ satisfying $0\leq \zeta_{B}\left(x\right)+\varrho_{B}\left(x\right)+\gamma_{B}\left(x\right)\leq 1$ and $0\leq \zeta_{A}\left(x\right)+\varrho_{A}\left(x\right)+\gamma_{A}\left(x\right)\leq 1$. Thus, $\left(Y_{1},A\right)⋒\left(Y_{2},B\right)=\left(Y_{2},B\right)⋒\left(Y_{1},A\right)$.
2. It follows easily from part (1).

□

**Definition** **14.** *Let (Y,A) and (Ω,B) be two PFSESs over X. Then operation ‘AND’ between them is represented as $\left(Y,A\right)\bar{\wedge }\left(\Omega ,B\right)$ and is given by $\left(Y,A\right)\bar{\wedge }\left(\Omega ,B\right)=\left(ϝ,A\times B\right)$ where ϝ(α,β)=Y(α)∩Ω(β), which is defined as:*

$$ϝ\left(\alpha ,\beta \right)\left(x\right)=\{\min\left(\zeta_{A}\left(x\right),\zeta_{B}\left(x\right)\right),\min\left(\varrho_{A}\left(x\right),\varrho_{B}\left(x\right)\right),\max\left(\gamma_{A}\left(x\right),\gamma_{B}\left(x\right)\right)\},$$

∀ (α,β)∈A×B, x∈X.

**Example** **8.** *Reconsider data in Example 1 and let (Y,A) and (Ω,B) be PFSESs over X, which are provided by Table 11 and Table 12, respectively, where A and B are given as below:*

$$\begin{array}{c} A=\{\left(q_{1},e_{3},1\right),\left(q_{2},e_{1},1\right),\left(q_{3},e_{2},0\right),\left(q_{4},e_{1},0\right)\}\\ B=\{\left(q_{1},e_{2},1\right),\left(q_{2},e_{2},1\right),\left(q_{3},e_{1},0\right),\left(q_{4},e_{3},0\right)\} \end{array}$$

*Then the operation AND between PFSESs (Y,A) and (Ω,B) is given in Table 13.*

**Definition** **15.** *Let (Y,A) and (Ω,B) be two PFSESs over X. Then the operation ‘OR’ between PFSESs (Y,A) and (Ω,B) is represented by $\left(Y,A\right)\underline{\vee }\left(\Omega ,B\right)$ and is given as $\left(Y,A\right)\underline{\vee }\left(\Omega ,B\right)=\left(\Theta ,A\times B\right)$ where Θ(α,β)=Y(α)∩Ω(β), which is defined as:*

$$\Theta \left(\alpha ,\beta \right)\left(x\right)=\{\max\left(\zeta_{A}\left(x\right),\zeta_{B}\left(x\right)\right),\min\left(\varrho_{A}\left(x\right),\varrho_{B}\left(x\right)\right),\min\left(\gamma_{A}\left(x\right),\gamma_{B}\left(x\right)\right)\},$$

*for all (α,β)∈A×B and x∈X.*

**Example** **9.** *Reconsider PFSESs (Y,A) and (Ω,B), which are provided by Table 11 and Table 12, respectively. Then, the ‘OR operation’ between them is given by Table 14.*

**Proposition** **4.** *Let $\left(Y_{1},A\right)$ and $\left(Y_{2},B\right)$ be PFSESs on X. Then*

1.
${\left(\left(Y_{1},A\right)\bar{\wedge }\left(Y_{2},B\right)\right)}^{c}={\left(Y_{1},A\right)}^{c}\underline{\vee }{\left(Y_{2},B\right)}^{c},$

2.
${\left(\left(Y_{1},A\right)\underline{\vee }\left(Y_{2},B\right)\right)}^{c}={\left(Y_{1},A\right)}^{c}\bar{\wedge }{\left(Y_{2},B\right)}^{c}.$

**Proof**. Its proof follows from Definitions 14 and 15.  □

**Proposition** **5.** *Let $\left(Y_{1},A\right)$, $\left(Y_{2},B\right)$ and $\left(Y_{3},C\right)$ be PFSESs over X. Then*

*1*.
$\left(Y_{1},A\right)\bar{\wedge }\left(\left(Y_{2},B\right)\bar{\wedge }\left(Y_{3},C\right)\right)=\left(\left(Y_{1},A\right)\bar{\wedge }\left(Y_{2},B\right)\right)\bar{\wedge }\left(Y_{3},C\right)$ *,*
*2*.
$\left(Y_{1},A\right)\underline{\vee }\left(\left(Y_{2},B\right)\underline{\vee }\left(Y_{3},C\right)\right)=\left(\left(Y_{1},A\right)\underline{\vee }\left(Y_{2},B\right)\right)\underline{\vee }\left(Y_{3},C\right)$ *,*
*3*.
$\left(Y_{1},A\right)\bar{\wedge }\left(\left(Y_{2},B\right)\underline{\vee }\left(Y_{3},C\right)\right)=\left(\left(Y_{1},A\right)\bar{\wedge }\left(Y_{2},B\right)\right)\underline{\vee }\left(\left(Y_{1},A\right)\bar{\wedge }\left(Y_{3},C\right)\right)$ *,*
*4*.
$\left(Y_{1},A\right)\underline{\vee }\left(\left(Y_{2},B\right)\bar{\wedge }\left(Y_{3},C\right)\right)=\left(\left(Y_{1},A\right)\underline{\vee }\left(Y_{2},B\right)\right)\bar{\wedge }\left(\left(Y_{1},A\right)\underline{\vee }\left(Y_{3},C\right)\right)$ *.*

**Proof.**  Using Definition 14,

$$\left(Y_{1},A\right)\bar{\wedge }\left(\left(Y_{2},B\right)\bar{\wedge }\left(Y_{3},C\right)\right)=\left(ϝ,A\times \left(B\times C\right)\right)$$

where $ϝ\left(\varphi_{1},\left(\varphi_{2},\varphi_{3}\right)\right)=Y_{1}\left(\varphi_{1}\right)\cap \left(Y_{2}\left(\varphi_{2}\right)\cap Y_{3}\left(\varphi_{3}\right)\right),$ for all $\left(\varphi_{1},\left(\varphi_{2},\varphi_{3}\right)\right)\in A\times \left(B\times C\right)$. Using the Proposition 3, we obtain $Y_{1}\left(\varphi_{1}\right)\cap \left(Y_{2}\left(\varphi_{2}\right)\cap Y_{3}\left(\varphi_{3}\right)\right)=\left(Y_{1}\left(\varphi_{1}\right)\cap Y_{2}\left(\varphi_{2}\right)\right)\cap Y_{3}\left(\varphi_{3}\right)$. Thus, $\left(Y_{1},A\right)\bar{\wedge }\left(\left(Y_{2},B\right)\bar{\wedge }\left(Y_{3},C\right)\right)=\left(\left(Y_{1},A\right)\bar{\wedge }\left(Y_{2},B\right)\right)\bar{\wedge }\left(Y_{3},C\right)$.

The remaining parts followed similarly as part (1).

□

## 4. Application to Group Decision Making

Virtual reality (VR) originated in 1957 by Morton Heilig. His interactive media gadget called the Sensorama is regarded one of the foremost VR systems. Actually, the term ‘virtual reality’ or VR was introduced in 1987 by analyzer Jaron Lanier, whose analysis and work promoted several areas of the VR industry. The technology of VR basically depends on the usage of computer engineering to generate a simulated circumstance. As a replacement for seeing a screen in front of the users, they are immersed and able to interconnect with a three-dimensional (3D) world. By simulating as many senses as achievable, namely, hearing, touch, sight and even smell, the computer is converted into a doorkeeper to this unnatural world. VR’s most instantly identifiable element is the head-mounted display. There are many important scientific fields where VR is playing an outstanding role such as VR in healthcare, VR in education and VR in military. VR is utilized in multiple sectors of healthcare. Any type of medical circumstance can be simulated using VR, to permit the students to manage with it as in actual life. VR can be employed to improve student learning abilities. VR education can efficiently modify the process by which educational content is provided; it operates on the basis of producing a virtual real world or allows users to not only watch it but also interact with it. In the late 1920s and 1930s, flight trainers from the Link Company was the earliest utilization of simulators in a military environment. For training purposes, VR has also been used by military forces, including army, navy and air force. Further, the entertainment industry is one of the most enthusiastic applications of VR. At the beginning of 2017, the British Museum presented a VR experience of the British Museum, offering users an unparalleled digital experience by mobile device or using computer, and the opportunity to be completely engaged with a VR headset. The above discussion reveals an important fact that the selection of the best VR system manufacturing company is an uncertain problem due to multiple characteristics (parameters) of VR systems. So, it is a critical task for the buyers to choose the best option. The selection of an appropriate company manufacturing VR systems is possible with the help of different experts’ evaluations about VR systems according to the favorable parameters of buyers (wholesale dealers).

Suppose $X=\{x_{1}=\mathrm{Applied}\;\mathrm{VR},x_{2}=\mathrm{Phaser}\;\mathrm{Lock}\;\mathrm{Interactive},x_{3}=\mathrm{Lucid}\;\mathrm{Sight},$$x_{4}=\mathrm{Owlchemy}\;\mathrm{Labs},x_{5}=\mathrm{WEVR},x_{6}=\mathrm{Unity}\;\mathrm{Technologies}\}$ is the set of well-known companies that are producing VR systems. In order to obtain the best VR system producing company, consider $Q=\{q_{1},q_{2},\ldots ,q_{12}\}$ is the set of parameters used by a dealer for the selection of best company regarding the production of VR systems where

- $q_{1}$ serves as interaction,
- $q_{2}$ serves as video games,
- $q_{3}$ serves as education,
- $q_{4}$ serves as sensory feedback,
- $q_{5}$ serves as training,
- $q_{6}$ serves as effective communication,
- $q_{7}$ serves as convenience,
- $q_{8}$ serves as comfort,
- $q_{9}$ serves as building student skills,
- $q_{10}$ serves as detail views,
- $q_{11}$ serves as connect with people,
- $q_{12}$ serves as realistic.

Assume that $E=\{e_{1},e_{2},e_{3}\}$ is a collection of three experts invited by the dealer to determine the most suitable company regarding manufacturing of VR systems and OP={1,0} is the set of opinions where 1=agree and 0=disagree. Suppose that experts provide their judgments in the form of a PFSES (Y,A) where A⊆S=Q×E×OP. For simplicity, PFSES (Y,A) is divided into agree and disagree components, respectively. In the following, Table 15 displays the agree-PFSES while Table 16 represents the disagree-PFSES.

Now by using Definition 4, the score values of both agree-PFSES and disagree-PFSES are computed in Table 17 and Table 18, respectively. Moreover, accumulative score values are also determined in the last rows of these tables.

We now provide an algorithm that will be helpful in the selection process for the best option under the picture fuzzy soft expert framework (see Algorithm 1).
**Algorithm 1:** Selection of an appropriate option under PFSESs**Input:**X, the universal set with *n* elementsQ, the set of parametersE, the set of expertsOP, the set of opinionsFor A⊆S, where S=Q×E×OP, insert a PFSES (Y,A) based on the evaluations of invited experts.Compute an agree-PFSES ${\left(Y,A\right)}_{1}$ and disagree-PFSES ${\left(Y,A\right)}_{0}$.By Definition 4, determine score values for both agree ${\left(Y,A\right)}_{1}$ and disagree ${\left(Y,A\right)}_{0}$ components of PFSES.Calculate accumulative scores $a_{j}=\sum_{i}e_{ij}$ in the score table of agree-PFSES ${\left(Y,A\right)}_{1}$.Calculate accumulative scores $b_{j}=\sum_{i}e_{ij}$ in the score table of disagree-PFSES ${\left(Y,A\right)}_{0}$.Determine $z_{j}=a_{j}-b_{j},\;j=1,2,\ldots ,n$.Find *r*, for which $z_{r}=\max z_{j}$.**Output:** The object having maximum final score value in step ‘7’ will be the decision. In case if two or more values have similar final score then any one of them can be chosen as decision object.

By applying the above algorithm, the final score values are computed in the following Table 19.

From Table 19, it can easily seen that the best choice for the dealer to select a virtual reality system producing company is $x_{4}$; therefore, the dealer will select company $x_{4}$ for his consignment order of VR systems.

**Figure 1:**
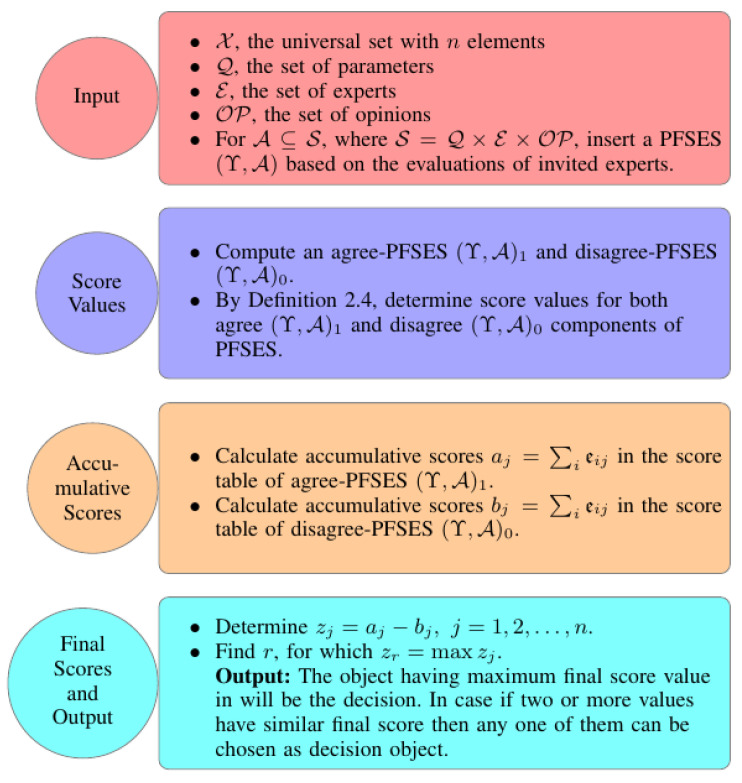
Flowchart diagram.

To better understand the initiated algorithmic approach, its flowchart diagram is provided in Figure 1.

## 5. Discussion

With the analysis of the last few decades, from the invention of FS theory to present day, we can easily observe that a number of research scholars from almost every domain of science put themselves into a race of producing different natural generalizations of FSs, such as IFSs, bipolar FSs, interval-valued FSs, PFSs, etc., or hybrid models of these extensions with other existing uncertainty theories, including soft sets, rough sets and SESs. One can easily notice that hybrid models of PFS with SES are still unable to handle picture fuzzy soft information more efficiently with ‘t’ experts, t>1. Motivated by this fact, in this study, a novel hybrid model called PFSESs is proposed by the combination of above-mentioned existing theories. Our developed model has the ability to deal with the evaluations of more than one expert regarding each alternative with respect to each parameter under consideration. The proposed approach is very reliable and feasible for dealing with imprecise and vague picture fuzzy soft expert information. Particularly, when the problem under consideration is based on picture fuzzy soft information collected with the judgments of different experts.

**Figure 2:**
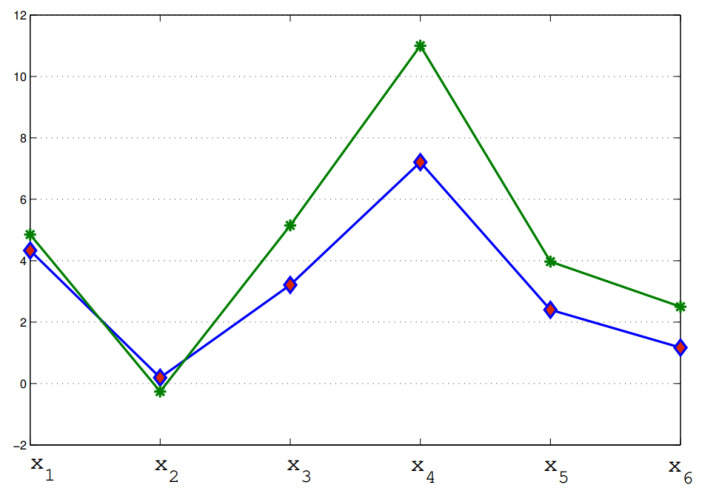
Comparison of proposed PFSES model with IFSESs [38].

Many fruitful results have been produced as an extension of PFSs, such as PFSSs, to handle different problems of several scientific fields, including artificial intelligence. Since PFSS is a soft extension of the PFS model but fails to deal with the individual evaluation of more than one expert in a group decision-making situation, we created our proposed model as an efficient generalization of SESs, FSESs, IFSESs or PFSSs. We have checked the effectiveness of our proposed model by solving the application in Section 4 with the proposed approach and existing IFSES model. Clearly, we obtain optimal results; however, there is a minor change in their ranking order of sub-optimal decision objects. We provide the comparative analysis of the developed PFSES model in both qualitative and quantitative modes, which are displayed in Table 20 and Table 21 and Figure 2. We conclude that the initiated approach is more cogent and feasible to solve different real-world problems in the picture fuzzy soft expert environment.

### Limitations of the Initiated Model

In the past two decades, the impact of uncertainty theories in mathematical modeling-based real-world systems has increased substantially. A hybrid model is a combination of two or more theories that experts can use to make decisions. A good hybrid model has the ability to overcome some issue in existing theories and provide more accurate results than existing ones; however, it does not matter how good it is because models will almost always have limitations. In the following, we discuss the limitations of the initiated approach that we observed during its construction process.

- The initiated model fails to address a situation involving three-dimensional information where membership value is 0.4, non-membership value is 0.7 and neutral value is 0.1. Clearly, 0.4+0.7+0.1=1.2≮1.
- Since mathematical modeling is mainly dependent on the input data and evaluations. The speed of the proposed hybrid model regarding computation may be slow in the case of a large data-set. This deficiency is present in almost every existing model that can be overcome via an appropriate coding method with the help of software, including MATLAB.
- Another difficulty of our initiated model is the change in rank of alternatives if existing parameters (or alternatives) are deleted or new parameters (or alternatives) are inserted in a group decision-making problem. The main reason behind these problems is the independent behavior of objects and parameters.

## 6. Conclusions

Group decision-making issues are of great significance in various fields ranging from medicine to engineering. To cope with such issues, PFSs are becoming a generic mathematical tool for handling imprecision and vagueness in different group decision-making situations. A noticeable growth is found regarding the use of PFSs and PFSSs in real-world decision-making issues. The major goal of this study is to present a new hybrid model, called PFSES, which is an extension of PFSS or FSES or IFSES. This novel concept can be employed to describe picture fuzzy knowledge in a more effective and general manner. Specifically, some fundamental operations, such as subset, equality, union, intersection, complement, OR operation, AND operation and agree- and disagree-PFSESs, are developed and are investigated with respective numerical examples. Further, certain De Morgan’s laws for PFSESs are verified with respect to the AND and OR operations. Moreover, a method is presented to solve the MAGDM problems based upon the PFSES framework. Our proposed methodology is tested on a practical application to describe the validity and cogency of the developed hybrid model, that is, a selection of best VR system manufacturing company. Finally, a comparison of the presented approach with some existing models, including IFSESs [38] is provided. From Figure 2 and Table 20, we have observed that the optimal decision object is the same ($x_{4}$) by solving the proposed application with both IFSESs and the initiated PFSES model. Thus, as an extension of PFSSs regarding experts, the presented approach for MAGDM is very feasible and more reliable than existing SES models. In future work, we are trying to extend our study with the following models:

1. *q*-Rung orthopair picture fuzzy soft expert sets,
2. Interval-valued picture fuzzy soft expert sets,
3. Fuzzy parameterized picture fuzzy soft expert sets.

## Figures and Tables

**Table 1:** Tabular form of the PFSES (Y,A).

(Y,A)	$x_{1}$	…	$x_{n}$
$\left(q_{1},e_{1},1\right)$	$\left(\zeta_{A}\left(q_{1},e_{1},1\right),\varrho_{A}\left(q_{1},e_{1},1\right),\gamma_{A}\left(q_{1},e_{1},1\right)\right)$	…	$\left(\zeta_{A}\left(q_{1},e_{1},1\right),\varrho_{A}\left(q_{1},e_{1},1\right),\gamma_{A}\left(q_{1},e_{1},1\right)\right)$
$\left(q_{1},e_{2},1\right)$	$\left(\zeta_{A}\left(q_{1},e_{2},1\right),\varrho_{A}\left(q_{1},e_{2},1\right),\gamma_{A}\left(q_{1},e_{2},1\right)\right)$	…	$\left(\zeta_{A}\left(q_{1},e_{2},1\right),\varrho_{A}\left(q_{1},e_{2},1\right),\gamma_{A}\left(q_{1},e_{2},1\right)\right)$
⋮	⋮	⋱	⋮
$\left(q_{m},e_{t},1\right)$	$\left(\zeta_{A}\left(q_{m},e_{t},1\right),\varrho_{A}\left(q_{m},e_{t},1\right),\gamma_{A}\left(q_{m},e_{t},1\right)\right)$	…	$\left(\zeta_{A}\left(q_{m},e_{t},1\right),\varrho_{A}\left(q_{m},e_{t},1\right),\gamma_{A}\left(q_{m},e_{t},1\right)\right)$
$\left(q_{1},e_{1},0\right)$	$\left(\zeta_{A}\left(q_{1},e_{1},0\right),\varrho_{A}\left(q_{1},e_{1},0\right),\gamma_{A}\left(q_{1},e_{1},0\right)\right)$	…	$\left(\zeta_{A}\left(q_{1},e_{1},0\right),\varrho_{A}\left(q_{1},e_{1},0\right),\gamma_{A}\left(q_{1},e_{1},0\right)\right)$
$\left(q_{1},e_{2},0\right)$	$\left(\zeta_{A}\left(q_{1},e_{2},0\right),\varrho_{A}\left(q_{1},e_{2},0\right),\gamma_{A}\left(q_{1},e_{2},0\right)\right)$	…	$\left(\zeta_{A}\left(q_{1},e_{2},0\right),\varrho_{A}\left(q_{1},e_{2},0\right),\gamma_{A}\left(q_{1},e_{2},0\right)\right)$
⋮	⋮	⋱	⋮
$\left(q_{m},e_{t},0\right)$	$\left(\zeta_{A}\left(q_{m},e_{t},0\right),\varrho_{A}\left(q_{m},e_{t},0\right),\gamma_{A}\left(q_{m},e_{t},0\right)\right)$	…	$\left(\zeta_{A}\left(q_{m},e_{t},0\right),\varrho_{A}\left(q_{m},e_{t},0\right),\gamma_{A}\left(q_{m},e_{t},0\right)\right)$

**Table 2:** Tabular representation of the PFSES (Y,A).

(Y,A)	$x_{1}$	$x_{2}$	$x_{3}$	$x_{4}$	$x_{5}$	$x_{6}$
$\left(q_{1},e_{1},1\right)$	(0.4,0.2,0.2)	(0.3,0.2,0.1)	(0.1,0.2,0.5)	(0.6,0.2,0.1)	(0.3,0.5,0.1)	(0.4,0.3,0.2)
$\left(q_{1},e_{2},1\right)$	(0.5,0.3,0.1)	(0.6,0.1,0.1)	(0.1,0.5,0.1)	(0.5,0.2,0.2)	(0.2,0.4,0.2)	(0.2,0.3,0.4)
$\left(q_{1},e_{3},1\right)$	(0.7,0.1,0.1)	(0.4,0.2,0.2)	(0.5,0.1,0.3)	(0.3,0.3,0.2)	(0.7,0.1,0.2)	(0.1,0.5,0.3)
$\left(q_{2},e_{1},1\right)$	(0.2,0.2,0.3)	(0.2,0.1,0.5)	(0.2,0.3,0.4)	(0.3,0.1,0.2)	(0.5,0.3,0.1)	(0.5,0.2,0.3)
$\left(q_{2},e_{2},1\right)$	(0.3,0.3,0.4)	(0.2,0.4,0.1)	(0.4,0.2,0.1)	(0.4,0.4,0.2)	(0.2,0.3,0.4)	(0.2,0.1,0.3)
$\left(q_{2},e_{3},1\right)$	(0.6,0.2,0.2)	(0.2,0.4,0.1)	(0.6,0.1,0.1)	(0.2,0.5,0.2)	(0.2,0.3,0.4)	(0.6,0.1,0.1)
$\left(q_{3},e_{1},1\right)$	(0.4,0.3,0.2)	(0.2,0.05,0.1)	(0.09,0.01,0.1)	(0.3,0.05,0.2)	(0.2,0.05,0.06)	(0.6,0.3,0.1)
$\left(q_{3},e_{2},1\right)$	(0.5,0.09,0.2)	(0.6,0.05,0.1)	(0.04,0.02,0.4)	(0.5,0.05,0.1)	(0.3,0.02,0.03)	(0.5,0.3,0.1)
$\left(q_{3},e_{3},1\right)$	(0.7,0.1,0.02)	(0.3,0.04,0.01)	(0.08,0.1,0.1)	(0.4,0.09,0.2)	(0.2,0.5,0.09)	(0.07,0.3,0.1)
$\left(q_{4},e_{1},1\right)$	(0.6,0.3,0.1)	(0.09,0.3,0.1)	(0.04,0.7,0.1)	(0.6,0.04,0.02)	(0.5,0.03,0.04)	(0.1,0.04,0.3)
$\left(q_{4},e_{2},1\right)$	(0.05,0.02,0.3)	(0.03,0.5,0.1)	(0.3,0.2,0.04)	(0.05,0.02,0.1)	(0.4,0.06,0.01)	(0.4,0.03,0.2)
$\left(q_{4},e_{3},1\right)$	(0.08,0.04,0.1)	(0.06,0.4,0.3)	(0.2,0.03,0.01)	(0.4,0.07,0.2)	(0.09,0.2,0.1)	(0.3,0.2,0.1)
$\left(q_{1},e_{1},0\right)$	(0.05,0.09,0.3)	(0.08,0.06,0.2)	(0.09,0.2,0.3)	(0.05,0.5,0.01)	(0.04,0.1,0.2)	(0.5,0.1,0.1)
$\left(q_{1},e_{2},0\right)$	(0.06,0.5,0.2)	(0.08,0.4,0.2)	(0.05,0.4,0.2)	(0.4,0.3,0.2)	(0.05,0.4,0.1)	(0.2,0.3,0.2)
$\left(q_{1},e_{3},0\right)$	(0.08,0.07,0.2)	(0.09,0.04,0.4)	(0.07,0.08,0.3)	(0.05,0.07,0.6)	(0.04,0.07,0.3)	(0.03,0.4,0.2)
$\left(q_{2},e_{1},0\right)$	(0.6,0.2,0.1)	(0.5,0.3,0.2)	(0.09,0.3,0.3)	(0.08,0.4,0.3)	(0.07,0.6,0.3)	(0.07,0.6,0.3)
$\left(q_{2},e_{2},0\right)$	(0.5,0.08,0.2)	(0.09,0.5,0.3)	(0.08,0.6,0.4)	(0.5,0.06,0.3)	(0.08,0.7,0.1)	(0.03,0.2,0.4)
$\left(q_{2},e_{3},0\right)$	(0.5,0.08,0.2)	(0.09,0.5,0.3)	(0.08,0.6,0.3)	(0.5,0.06,0.3)	(0.8,0.07,0.1)	(0.4,0.01,0.3)
$\left(q_{3},e_{1},0\right)$	(0.05,0.07,0.1)	(0.06,0.08,0.2)	(0.08,0.4,0.4)	(0.09,0.1,0.2)	(0.7,0.06,0.1)	(0.3,0.04,0.1)
$\left(q_{3},e_{2},0\right)$	(0.08,0.3,0.2)	(0.05,0.4,0.2)	(0.06,0.08,0.2)	(0.05,0.06,0.4)	(0.09,0.3,0.5)	(0.03,0.3,0.2)
$\left(q_{3},e_{3},0\right)$	(0.3,0.1,0.4)	(0.06,0.6,0.4)	(0.3,0.4,0.1)	(0.08,0.4,0.2)	(0.2,0.4,0.2)	(0.2,0.4,0.3)
$\left(q_{4},e_{1},0\right)$	(0.05,0.4,0.5)	(0.08,0.05,0.1)	(0.07,0.2,0.3)	(0.5,0.06,0.4)	(0.4,0.3,0.1)	(0.2,0.3,0.3)
$\left(q_{4},e_{2},0\right)$	(0.08,0.04,0.3)	(0.3,0.03,0.2)	(0.6,0.2,0.1)	(0.7,0.1,0.1)	(0.5,0.2,0.2)	(0.4,0.3,0.2)
$\left(q_{4},e_{3},0\right)$	(0.4,0.5,0.1)	(0.4,0.3,0.3)	(0.5,0.2,0.1)	(0.5,0.1,0.3)	(0.2,0.1,0.4)	(0.2,0.4,0.3)

**Table 3:** Tabular representation of the PFSES (Y,A).

(Y,A)	$x_{1}$	$x_{2}$	$x_{3}$	$x_{4}$	$x_{5}$	$x_{6}$
$\left(q_{1},e_{2},1\right)$	(0.5,0.3,0.1)	(0.6,0.1,0.1)	(0.1,0.5,0.1)	(0.5,0.2,0.2)	(0.2,0.4,0.2)	(0.2,0.3,0.4)
$\left(q_{2},e_{1},1\right)$	(0.2,0.2,0.3)	(0.2,0.1,0.5)	(0.2,0.3,0.4)	(0.3,0.1,0.2)	(0.5,0.3,0.1)	(0.5,0.2,0.3)
$\left(q_{3},e_{1},1\right)$	(0.4,0.3,0.2)	(0.2,0.05,0.1)	(0.09,0.01,0.1)	(0.3,0.05,0.2)	(0.2,0.05,0.06)	(0.6,0.3,0.1)
$\left(q_{4},e_{3},1\right)$	(0.08,0.04,0.1)	(0.06,0.4,0.3)	(0.2,0.03,0.01)	(0.4,0.07,0.2)	(0.09,0.2,0.1)	(0.3,0.2,0.1)
$\left(q_{1},e_{3},0\right)$	(0.08,0.07,0.2)	(0.09,0.04,0.4)	(0.07,0.08,0.3)	(0.05,0.07,0.6)	(0.04,0.07,0.3)	(0.03,0.4,0.2)
$\left(q_{2},e_{3},0\right)$	(0.5,0.08,0.2)	(0.09,0.5,0.3)	(0.08,0.6,0.3)	(0.5,0.06,0.3)	(0.8,0.07,0.1)	(0.4,0.01,0.3)
$\left(q_{3},e_{2},0\right)$	(0.08,0.3,0.2)	(0.05,0.4,0.2)	(0.06,0.08,0.2)	(0.05,0.06,0.4)	(0.09,0.3,0.5)	(0.03,0.3,0.2)
$\left(q_{4},e_{1},0\right)$	(0.05,0.4,0.5)	(0.08,0.05,0.1)	(0.07,0.2,0.3)	(0.5,0.06,0.4)	(0.4,0.3,0.1)	(0.2,0.3,0.3)

**Table 4:** Tabular representation of the PFSES (Ω,B).

(Ω,B)	$x_{1}$	$x_{2}$	$x_{3}$	$x_{4}$	$x_{5}$	$x_{6}$
$\left(q_{1},e_{2},1\right)$	(0.6,0.3,0.1)	(0.7,0.2,0.1)	(0.2,0.6,0.1)	(0.6,0.3,0.01)	(0.4,0.5,0.02)	(0.4,0.3,0.05)
$\left(q_{2},e_{1},1\right)$	(0.4,0.3,0.1)	(0.4,0.2,0.05)	(0.3,0.5,0.04)	(0.5,0.3,0.1)	(0.6,0.3,0.01)	(0.7,0.2,0.03)
$\left(q_{3},e_{1},1\right)$	(0.5,0.4,0.03)	(0.4,0.1,0.05)	(0.2,0.4,0.09)	(0.5,0.1,0.03)	(0.4,0.1,0.02)	(0.6,0.3,0.01)
$\left(q_{4},e_{3},1\right)$	(0.3,0.2,0.07)	(0.4,0.3,0.05)	(0.5,0.2,0.01)	(0.5,0.2,0.01)	(0.3,0.4,0.01)	(0.4,0.3,0.05)
$\left(q_{1},e_{3},0\right)$	(0.2,0.3,0.05)	(0.4,0.2,0.07)	(0.4,0.3,0.04)	(0.3,0.5,0.08)	(0.3,0.2,0.1)	(0.4,0.5,0.06)
$\left(q_{2},e_{3},0\right)$	(0.6,0.1,0.09)	(0.3,0.6,0.01)	(0.1,0.7,0.06)	(0.7,0.1,0.08)	(0.9,0.08,0.02)	(0.5,0.06,0.04)
$\left(q_{3},e_{2},0\right)$	(0.2,0.4,0.01)	(0.3,0.5,0.03)	(0.4,0.2,0.08)	(0.5,0.2,0.08)	(0.4,0.5,0.08)	(0.5,0.4,0.1)
$\left(q_{4},e_{1},0\right)$	(0.3,0.5,0.07)	(0.3,0.5,0.09)	(0.4,0.5,0.2)	(0.6,0.3,0.04)	(0.5,0.4,0.01)	(0.3,0.4,0.09)

**Table 5:** Tabular representation of an agree-PFSES ${\left(Y,A\right)}_{1}$.

${\left(Y,A\right)}_{1}$	$x_{1}$	$x_{2}$	$x_{3}$	$x_{4}$	$x_{5}$	$x_{6}$
$\left(q_{1},e_{1},1\right)$	(0.4,0.2,0.2)	(0.3,0.2,0.1)	(0.1,0.2,0.5)	(0.6,0.2,0.1)	(0.3,0.5,0.1)	(0.4,0.3,0.2)
$\left(q_{1},e_{2},1\right)$	(0.5,0.3,0.1)	(0.6,0.1,0.1)	(0.1,0.5,0.1)	(0.5,0.2,0.2)	(0.2,0.4,0.2)	(0.2,0.3,0.4)
$\left(q_{1},e_{3},1\right)$	(0.7,0.1,0.1)	(0.4,0.2,0.2)	(0.5,0.1,0.3)	(0.3,0.3,0.2)	(0.7,0.1,0.2)	(0.1,0.5,0.3)
$\left(q_{2},e_{1},1\right)$	(0.2,0.2,0.3)	(0.2,0.1,0.5)	(0.2,0.3,0.4)	(0.3,0.1,0.2)	(0.5,0.3,0.1)	(0.5,0.2,0.3)
$\left(q_{2},e_{2},1\right)$	(0.3,0.3,0.4)	(0.2,0.4,0.1)	(0.4,0.2,0.1)	(0.4,0.4,0.2)	(0.2,0.3,0.4)	(0.2,0.1,0.3)
$\left(q_{2},e_{3},1\right)$	(0.6,0.2,0.2)	(0.2,0.4,0.1)	(0.6,0.1,0.1)	(0.2,0.5,0.2)	(0.2,0.3,0.4)	(0.6,0.1,0.1)
$\left(q_{3},e_{1},1\right)$	(0.4,0.3,0.2)	(0.2,0.05,0.1)	(0.09,0.01,0.1)	(0.3,0.05,0.2)	(0.2,0.05,0.06)	(0.6,0.3,0.1)
$\left(q_{3},e_{2},1\right)$	(0.5,0.09,0.2)	(0.6,0.05,0.1)	(0.04,0.02,0.4)	(0.5,0.05,0.1)	(0.3,0.02,0.03)	(0.5,0.3,0.1)
$\left(q_{3},e_{3},1\right)$	(0.7,0.1,0.02)	(0.3,0.04,0.01)	(0.08,0.1,0.1)	(0.4,0.09,0.2)	(0.2,0.5,0.09)	(0.07,0.3,0.1)
$\left(q_{4},e_{1},1\right)$	(0.6,0.3,0.1)	(0.09,0.3,0.1)	(0.04,0.7,0.1)	(0.6,0.04,0.02)	(0.5,0.03,0.04)	(0.1,0.04,0.3)
$\left(q_{4},e_{2},1\right)$	(0.05,0.02,0.3)	(0.03,0.5,0.1)	(0.3,0.2,0.04)	(0.05,0.02,0.1)	(0.4,0.06,0.01)	(0.4,0.03,0.2)
$\left(q_{4},e_{3},1\right)$	(0.08,0.04,0.1)	(0.06,0.4,0.3)	(0.2,0.03,0.01)	(0.4,0.07,0.2)	(0.09,0.2,0.1)	(0.3,0.2,0.1)

**Table 6:** Tabular form of the disagree-PFSES ${\left(Y,A\right)}_{0}$.

${\left(Y,A\right)}_{0}$	$x_{1}$	$x_{2}$	$x_{3}$	$x_{4}$	$x_{5}$	$x_{6}$
$\left(q_{1},e_{1},0\right)$	(0.05,0.09,0.3)	(0.08,0.06,0.2)	(0.09,0.2,0.3)	(0.05,0.5,0.01)	(0.04,0.1,0.2)	(0.5,0.1,0.1)
$\left(q_{1},e_{2},0\right)$	(0.06,0.5,0.2)	(0.08,0.4,0.2)	(0.05,0.4,0.2)	(0.4,0.3,0.2)	(0.05,0.4,0.1)	(0.2,0.3,0.2)
$\left(q_{1},e_{3},0\right)$	(0.08,0.07,0.2)	(0.09,0.04,0.4)	(0.07,0.08,0.3)	(0.05,0.07,0.6)	(0.04,0.07,0.3)	(0.03,0.4,0.2)
$\left(q_{2},e_{1},0\right)$	(0.6,0.2,0.1)	(0.5,0.3,0.2)	(0.09,0.3,0.3)	(0.08,0.4,0.3)	(0.07,0.6,0.3)	(0.07,0.6,0.3)
$\left(q_{2},e_{2},0\right)$	(0.5,0.08,0.2)	(0.09,0.5,0.3)	(0.08,0.6,0.4)	(0.5,0.06,0.3)	(0.08,0.7,0.1)	(0.03,0.2,0.4)
$\left(q_{2},e_{3},0\right)$	(0.5,0.08,0.2)	(0.09,0.5,0.3)	(0.08,0.6,0.3)	(0.5,0.06,0.3)	(0.8,0.07,0.1)	(0.4,0.01,0.3)
$\left(q_{3},e_{1},0\right)$	(0.05,0.07,0.1)	(0.06,0.08,0.2)	(0.08,0.4,0.4)	(0.09,0.1,0.2)	(0.7,0.06,0.1)	(0.3,0.04,0.1)
$\left(q_{3},e_{2},0\right)$	(0.08,0.3,0.2)	(0.05,0.4,0.2)	(0.06,0.08,0.2)	(0.05,0.06,0.4)	(0.09,0.3,0.5)	(0.03,0.3,0.2)
$\left(q_{3},e_{3},0\right)$	(0.3,0.1,0.4)	(0.06,0.6,0.4)	(0.3,0.4,0.1)	(0.08,0.4,0.2)	(0.2,0.4,0.2)	(0.2,0.4,0.3)
$\left(q_{4},e_{1},0\right)$	(0.05,0.4,0.5)	(0.08,0.05,0.1)	(0.07,0.2,0.3)	(0.5,0.06,0.4)	(0.4,0.3,0.1)	(0.2,0.3,0.3)
$\left(q_{4},e_{2},0\right)$	(0.08,0.04,0.3)	(0.3,0.03,0.2)	(0.6,0.2,0.1)	(0.7,0.1,0.1)	(0.5,0.2,0.2)	(0.4,0.3,0.2)
$\left(q_{4},e_{3},0\right)$	(0.4,0.5,0.1)	(0.4,0.3,0.3)	(0.5,0.2,0.1)	(0.5,0.1,0.3)	(0.2,0.1,0.4)	(0.2,0.4,0.3)

**Table 7:** Tabular form of the complement of PFSESs (Y,A).

${\left(Y,A\right)}^{c}$	$x_{1}$	$x_{2}$	$x_{3}$	$x_{4}$	$x_{5}$	$x_{6}$
$\left(q_{1},e_{1},1\right)$	(0.2,0.2,0.4)	(0.1,0.2,0.3)	(0.5,0.2,0.1)	(0.1,0.2,0.6)	(0.1,0.5,0.3)	(0.2,0.3,0.4)
$\left(q_{1},e_{2},1\right)$	(0.1,0.3,0.5)	(0.1,0.1,0.6)	(0.1,0.5,0.1)	(0.2,0.2,0.5)	(0.2,0.4,0.2)	(0.4,0.3,0.2)
$\left(q_{1},e_{3},1\right)$	(0.1,0.1,0.7)	(0.2,0.2,0.4)	(0.3,0.1,0.5)	(0.2,0.3,0.3)	(0.2,0.1,0.7)	(0.3,0.5,0.1)
$\left(q_{2},e_{1},1\right)$	(0.3,0.2,0.2)	(0.5,0.1,0.2)	(0.4,0.3,0.2)	(0.2,0.1,0.3)	(0.1,0.3,0.5)	(0.3,0.2,0.5)
$\left(q_{2},e_{2},1\right)$	(0.4,0.3,0.3)	(0.1,0.4,0.2)	(0.1,0.2,0.4)	(0.2,0.4,0.4)	(0.4,0.3,0.2)	(0.3,0.1,0.2)
$\left(q_{2},e_{3},1\right)$	(0.2,0.2,0.6)	(0.1,0.4,0.2)	(0.1,0.1,0.6)	(0.2,0.5,0.2)	(0.4,0.3,0.2)	(0.1,0.1,0.6)
$\left(q_{3},e_{1},1\right)$	(0.2,0.3,0.4)	(0.1,0.05,0.2)	(0.1,0.01,0.09)	(0.2,0.05,0.3)	(0.06,0.05,0.2)	(0.1,0.3,0.6)
$\left(q_{3},e_{2},1\right)$	(0.2,0.09,0.5)	(0.1,0.05,0.6)	(0.4,0.02,0.04)	(0.1,0.05,0.5)	(0.03,0.02,0.3)	(0.1,0.3,0.5)
$\left(q_{3},e_{3},1\right)$	(0.02,0.1,0.7)	(0.01,0.04,0.3)	(0.1,0.1,0.081)	(0.2,0.09,0.4)	(0.09,0.5,0.2)	(0.1,0.3,0.071)
$\left(q_{4},e_{1},1\right)$	(0.1,0.3,0.6)	(0.1,0.3,0.09)	(0.1,0.7,0.04)	(0.02,0.04,0.6)	(0.04,0.03,0.5)	(0.3,0.04,0.1)
$\left(q_{4},e_{2},1\right)$	(0.3,0.02,0.05)	(0.1,0.5,0.03)	(0.04,0.2,0.3)	(0.1,0.02,0.05)	(0.01,0.06,0.4)	(0.2,0.03,0.4)
$\left(q_{4},e_{3},1\right)$	(0.1,0.04,0.08)	(0.3,0.4,0.06)	(0.01,0.03,0.2)	(0.2,0.07,0.4)	(0.1,0.2,0.09)	(0.1,0.2,0.3)
$\left(q_{1},e_{1},0\right)$	(0.3,0.09,0.05)	(0.2,0.06,0.08)	(0.3,0.2,0.09)	(0.01,0.5,0.05)	(0.2,0.1,0.04)	(0.1,0.1,0.5)
$\left(q_{1},e_{2},0\right)$	(0.2,0.5,0.06)	(0.2,0.4,0.08)	(0.2,0.4,0.05)	(0.2,0.3,0.4)	(0.1,0.4,0.05)	(0.2,0.3,0.2)
$\left(q_{1},e_{3},0\right)$	(0.2,0.07,0.08)	(0.4,0.04,0.09)	(0.3,0.08,0.07)	(0.6,0.07,0.05)	(0.3,0.07,0.04)	(0.2,0.4,0.03)
$\left(q_{2},e_{1},0\right)$	(0.1,0.2,0.6)	(0.2,0.3,0.5)	(0.3,0.3,0.09)	(0.3,0.4,0.08)	(0.3,0.6,0.07)	(0.3,0.6,0.07)
$\left(q_{2},e_{2},0\right)$	(0.2,0.08,0.5)	(0.3,0.5,0.09)	(0.4,0.6,0.08)	(0.3,0.06,0.5)	(0.1,0.7,0.08)	(0.4,0.2,0.03)
$\left(q_{2},e_{3},0\right)$	(0.2,0.08,0.5)	(0.3,0.5,0.09)	(0.3,0.6,0.08)	(0.3,0.06,0.5)	(0.1,0.07,0.8)	(0.3,0.01,0.4)
$\left(q_{3},e_{1},0\right)$	(0.1,0.07,0.05)	(0.2,0.08,0.06)	(0.4,0.4,0.08)	(0.2,0.1,0.09)	(0.1,0.06,0.7)	(0.1,0.04,0.3)
$\left(q_{3},e_{2},0\right)$	(0.2,0.3,0.08)	(0.2,0.4,0.05)	(0.2,0.08,0.06)	(0.4,0.06,0.05)	(0.5,0.3,0.09)	(0.2,0.3,0.03)
$\left(q_{3},e_{3},0\right)$	(0.4,0.1,0.3)	(0.4,0.6,0.06)	(0.1,0.4,0.3)	(0.2,0.4,0.08)	(0.2,0.4,0.2)	(0.3,0.4,0.2)
$\left(q_{4},e_{1},0\right)$	(0.5,0.4,0.05)	(0.1,0.05,0.08)	(0.3,0.2,0.07)	(0.4,0.06,0.5)	(0.1,0.3,0.4)	(0.3,0.3,0.2)
$\left(q_{4},e_{2},0\right)$	(0.3,0.04,0.08)	(0.2,0.03,0.3)	(0.1,0.2,0.6)	(0.1,0.1,0.7)	(0.2,0.2,0.5)	(0.2,0.3,0.4)
$\left(q_{4},e_{3},0\right)$	(0.1,0.5,0.4)	(0.3,0.3,0.4)	(0.1,0.2,0.5)	(0.3,0.1,0.5)	(0.4,0.1,0.2)	(0.3,0.4,0.2)

**Table 8:** Tabular representation of the PFSESs (Ω,B).

(Ω,B)	$x_{1}$	$x_{2}$	$x_{3}$	$x_{4}$	$x_{5}$	$x_{6}$
$\left(q_{1},e_{1},1\right)$	(0.5,0.2,0.1)	(0.4,0.3,0.1)	(0.3,0.5,0.1)	(0.7,0.2,0.1)	(0.4,0.5,0.1)	(0.4,0.5,0.1)
$\left(q_{1},e_{2},1\right)$	(0.6,0.3,0.1)	(0.7,0.2,0.1)	(0.2,0.6,0.1)	(0.6,0.3,0.01)	(0.4,0.5,0.02)	(0.4,0.3,0.05)
$\left(q_{1},e_{3},1\right)$	(0.8,0.1,0.01)	(0.5,0.3,0.1)	(0.5,0.3,0.02)	(0.4,0.5,0.1)	(0.8,0.1,0.03)	(0.3,0.6,0.03)
$\left(q_{2},e_{1},1\right)$	(0.4,0.3,0.1)	(0.4,0.2,0.05)	(0.3,0.5,0.04)	(0.5,0.3,0.1)	(0.6,0.3,0.01)	(0.7,0.2,0.03)
$\left(q_{2},e_{2},1\right)$	(0.4,0.4,0.1)	(0.3,0.6,0.01)	(0.5,0.3,0.02)	(0.5,0.4,0.02)	(0.4,0.5,0.03)	(0.3,0.6,0.05)
$\left(q_{2},e_{3},1\right)$	(0.7,0.2,0.04)	(0.2,0.6,0.05)	(0.6,0.2,0.02)	(0.4,0.5,0.03)	(0.3,0.4,0.08)	(0.7,0.2,0.02)
$\left(q_{3},e_{1},1\right)$	(0.5,0.4,0.03)	(0.4,0.1,0.05)	(0.2,0.4,0.09)	(0.5,0.1,0.03)	(0.4,0.1,0.02)	(0.6,0.3,0.01)
$\left(q_{3},e_{2},1\right)$	(0.6,0.1,0.03)	(0.7,0.1,0.02)	(0.1,0.2,0.09)	(0.6,0.1,0.02)	(0.4,0.1,0.01)	(0.5,0.4,0.01)
$\left(q_{3},e_{3},1\right)$	(0.7,0.2,0.01)	(0.4,0.1,0.01)	(0.4,0.2,0.04)	(0.5,0.1,0.07)	(0.3,0.6,0.01)	(0.2,0.5,0.03)
$\left(q_{4},e_{1},1\right)$	(0.6,0.3,0.06)	(0.2,0.4,0.05)	(0.1,0.8,0.06)	(0.7,0.1,0.01)	(0.7,0.08,0.01)	(0.3,0.2,0.01)
$\left(q_{4},e_{2},1\right)$	(0.1,0.3,0.06)	(0.3,0.6,0.01)	(0.4,0.3,0.02)	(0.3,0.4,0.06)	(0.5,0.1,0.01)	(0.7,0.1,0.01)
$\left(q_{4},e_{3},1\right)$	(0.3,0.2,0.07)	(0.4,0.3,0.05)	(0.5,0.2,0.01)	(0.5,0.2,0.01)	(0.3,0.4,0.01)	(0.4,0.3,0.05)
$\left(q_{1},e_{1},0\right)$	(0.4,0.1,0.09)	(0.5,0.2,0.04)	(0.3,0.4,0.05)	(0.2,0.6,0.01)	(0.2,0.3,0.04)	(0.6,0.2,0.04)
$\left(q_{1},e_{2},0\right)$	(0.2,0.7,0.03)	(0.2,0.5,0.06)	(0.4,0.5,0.06)	(0.5,0.4,0.03)	(0.2,0.5,0.06)	(0.3,0.4,0.06)
$\left(q_{1},e_{3},0\right)$	(0.2,0.3,0.05)	(0.4,0.2,0.07)	(0.4,0.3,0.04)	(0.3,0.5,0.08)	(0.3,0.2,0.1)	(0.4,0.5,0.06)
$\left(q_{2},e_{1},0\right)$	(0.7,0.2,0.05)	(0.6,0.3,0.06)	(0.1,0.4,0.08)	(0.2,0.5,0.05)	(0.2,0.7,0.05)	(0.1,0.8,0.04)
$\left(q_{2},e_{2},0\right)$	(0.6,0.1,0.05)	(0.2,0.7,0.04)	(0.2,0.7,0.04)	(0.6,0.1,0.02)	(0.1,0.8,0.06)	(0.3,0.3,0.06)
$\left(q_{2},e_{3},0\right)$	(0.6,0.1,0.09)	(0.3,0.6,0.01)	(0.1,0.7,0.06)	(0.7,0.1,0.08)	(0.9,0.08,0.02)	(0.5,0.06,0.04)
$\left(q_{3},e_{1},0\right)$	(0.2,0.3,0.04)	(0.2,0.4,0.1)	(0.2,0.5,0.05)	(0.3,0.4,0.1)	(0.8,0.1,0.04)	(0.5,0.2,0.09)
$\left(q_{3},e_{2},0\right)$	(0.2,0.4,0.01)	(0.3,0.5,0.03)	(0.4,0.2,0.08)	(0.5,0.2,0.08)	(0.4,0.5,0.08)	(0.5,0.4,0.1)
$\left(q_{3},e_{3},0\right)$	(0.5,0.2,0.2)	(0.2,0.7,0.06)	(0.4,0.5,0.02)	(0.1,0.5,0.1)	(0.3,0.5,0.06)	(0.3,0.5,0.09)
$\left(q_{4},e_{1},0\right)$	(0.3,0.5,0.07)	(0.3,0.5,0.09)	(0.4,0.5,0.2)	(0.6,0.3,0.04)	(0.5,0.4,0.01)	(0.3,0.4,0.9)
$\left(q_{4},e_{2},0\right)$	(0.2,0.5,0.09)	(0.4,0.3,0.09)	(0.6,0.3,0.01)	(0.8,0.1,0.01)	(0.6,0.3,0.02)	(0.5,0.4,0.03)
$\left(q_{4},e_{3},0\right)$	(0.4,0.5,0.1)	(0.5,0.4,0.1)	(0.6,0.3,0.04)	(0.6,0.2,0.2)	(0.4,0.4,0.09)	(0.3,0.5,0.08)

**Table 9:** Tabular form of the union of PFSESs (Y,A) and (Ω,B).

(G,H)	$x_{1}$	$x_{2}$	$x_{3}$	$x_{4}$	$x_{5}$	$x_{6}$
$\left(q_{1},e_{1},1\right)$	(0.5,0.2,0.1)	(0.4,0.2,0.1)	(0.3,0.2,0.1)	(0.7,0.2,0.1)	(0.4,0.5,0.1)	(0.4,0.3,0.1)
$\left(q_{1},e_{2},1\right)$	(0.6,0.3,0.1)	(0.7,0.1,0.1)	(0.2,0.5,0.1)	(0.6,0.2,0.01)	(0.4,0.4,0.02)	(0.4,0.3,0.05)
$\left(q_{1},e_{3},1\right)$	(0.8,0.1,0.01)	(0.5,0.2,0.1)	(0.5,0.1,0.02)	(0.4,0.3,0.1)	(0.8,0.1,0.03)	(0.3,0.5,0.03)
$\left(q_{2},e_{1},1\right)$	(0.4,0.2,0.1)	(0.4,0.1,0.05)	(0.3,0.3,0.04)	(0.5,0.1,0.1)	(0.6,0.3,0.01)	(0.7,0.2,0.03)
$\left(q_{2},e_{2},1\right)$	(0.4,0.3,0.1)	(0.3,0.4,0.01)	(0.5,0.2,0.02)	(0.5,0.4,0.02)	(0.4,0.3,0.03)	(0.3,0.1,0.05)
$\left(q_{2},e_{3},1\right)$	(0.7,0.2,0.04)	(0.2,0.4,0.05)	(0.6,0.1,0.02)	(0.4,0.5,0.03)	(0.3,0.3,0.08)	(0.7,0.1,0.02)
$\left(q_{3},e_{1},1\right)$	(0.5,0.3,0.03)	(0.4,0.05,0.05)	(0.2,0.01,0.09)	(0.5,0.05,0.03)	(0.4,0.05,0.02)	(0.6,0.3,0.01)
$\left(q_{3},e_{2},1\right)$	(0.6,0.09,0.03)	(0.7,0.05,0.02)	(0.1,0.02,0.09)	(0.6,0.05,0.02)	(0.4,0.02,0.01)	(0.5,0.3,0.01)
$\left(q_{3},e_{3},1\right)$	(0.7,0.1,0.01)	(0.4,0.04,0.01)	(0.4,0.1,0.04)	(0.5,0.09,0.07)	(0.3,0.5,0.01)	(0.2,0.3,0.03)
$\left(q_{4},e_{1},1\right)$	(0.6,0.3,0.06)	(0.2,0.3,0.05)	(0.1,0.7,0.06)	(0.7,0.04,0.01)	(0.7,0.03,0.01)	(0.3,0.04,0.01)
$\left(q_{4},e_{2},1\right)$	(0.1,0.02,0.06)	(0.3,0.5,0.01)	(0.4,0.2,0.02)	(0.3,0.02,0.06)	(0.5,0.06,0.01)	(0.7,0.03,0.01)
$\left(q_{4},e_{3},1\right)$	(0.3,0.04,0.07)	(0.4,0.3,0.05)	(0.5,0.03,0.01)	(0.5,0.07,0.01)	(0.3,0.2,0.01)	(0.4,0.2,0.05)
$\left(q_{1},e_{1},0\right)$	(0.4,0.09,0.09)	(0.5,0.06,0.04)	(0.3,0.2,0.05)	(0.2,0.5,0.01)	(0.2,0.1,0.04)	(0.6,0.1,0.04)
$\left(q_{1},e_{2},0\right)$	(0.2,0.5,0.03)	(0.2,0.4,0.06)	(0.4,0.4,0.06)	(0.5,0.3,0.03)	(0.2,0.4,0.06)	(0.3,0.3,0.06)
$\left(q_{1},e_{3},0\right)$	(0.2,0.07,0.05)	(0.4,0.04,0.07)	(0.4,0.08,0.04)	(0.3,0.07,0.08)	(0.3,0.07,0.1)	(0.4,0.4,0.06)
$\left(q_{2},e_{1},0\right)$	(0.7,0.2,0.05)	(0.6,0.3,0.06)	(0.1,0.3,0.08)	(0.2,0.4,0.05)	(0.2,0.6,0.05)	(0.1,0.6,0.04)
$\left(q_{2},e_{2},0\right)$	(0.6,0.08,0.05)	(0.2,0.5,0.04)	(0.2,0.6,0.04)	(0.6,0.06,0.02)	(0.1,0.7,0.06)	(0.3,0.2,0.06)
$\left(q_{2},e_{3},0\right)$	(0.6,0.08,0.09)	(0.3,0.5,0.01)	(0.1,0.6,0.06)	(0.7,0.06,0.08)	(0.9,0.07,0.02)	(0.5,0.01,0.04)
$\left(q_{3},e_{1},0\right)$	(0.2,0.07,0.04)	(0.2,0.08,0.1)	(0.2,0.4,0.05)	(0.3,0.1,0.1)	(0.8,0.06,0.04)	(0.5,0.04,0.09)
$\left(q_{3},e_{2},0\right)$	(0.2,0.3,0.01)	(0.3,0.4,0.03)	(0.4,0.08,0.08)	(0.5,0.06,0.08)	(0.4,0.3,0.08)	(0.5,0.3,0.1)
$\left(q_{3},e_{3},0\right)$	(0.5,0.1,0.2)	(0.2,0.6,0.06)	(0.4,0.4,0.02)	(0.1,0.4,0.1)	(0.3,0.4,0.06)	(0.3,0.4,0.09)
$\left(q_{4},e_{1},0\right)$	(0.3,0.4,0.07)	(0.3,0.05,0.09)	(0.4,0.2,0.2)	(0.6,0.06,0.04)	(0.5,0.3,0.01)	(0.3,0.3,0.09)
$\left(q_{4},e_{2},0\right)$	(0.2,0.04,0.09)	(0.4,0.03,0.09)	(0.6,0.2,0.01)	(0.8,0.1,0.01)	(0.6,0.2,0.02)	(0.5,0.3,0.03)
$\left(q_{4},e_{3},0\right)$	(0.4,0.5,0.1)	(0.5,0.3,0.1)	(0.6,0.2,0.04)	(0.6,0.1,0.2)	(0.4,0.1,0.09)	(0.3,0.4,0.08)

**Table 10:** Tabular form of the intersection of PFSESs (Y,A) and (Ω,B).

(I,J)	$x_{1}$	$x_{2}$	$x_{3}$	$x_{4}$	$x_{5}$	$x_{6}$
$\left(q_{1},e_{1},1\right)$	(0.4,0.2,0.2)	(0.3,0.2,0.1)	(0.1,0.2,0.5)	(0.6,0.2,0.1)	(0.3,0.5,0.1)	(0.4,0.3,0.2)
$\left(q_{1},e_{2},1\right)$	(0.5,0.3,0.1)	(0.6,0.1,0.1)	(0.1,0.5,0.1)	(0.5,0.2,0.2)	(0.2,0.4,0.2)	(0.2,0.3,0.4)
$\left(q_{1},e_{3},1\right)$	(0.7,0.1,0.1)	(0.4,0.2,0.2)	(0.5,0.1,0.3)	(0.3,0.3,0.2)	(0.7,0.1,0.2)	(0.1,0.5,0.3)
$\left(q_{2},e_{1},1\right)$	(0.2,0.2,0.3)	(0.2,0.1,0.5)	(0.2,0.3,0.4)	(0.3,0.1,0.2)	(0.5,0.3,0.1)	(0.5,0.2,0.3)
$\left(q_{2},e_{2},1\right)$	(0.3,0.3,0.4)	(0.2,0.4,0.1)	(0.4,0.2,0.1)	(0.4,0.4,0.2)	(0.2,0.3,0.4)	(0.2,0.1,0.3)
$\left(q_{2},e_{3},1\right)$	(0.6,0.2,0.2)	(0.2,0.4,0.1)	(0.6,0.1,0.1)	(0.2,0.5,0.2)	(0.2,0.3,0.4)	(0.6,0.1,0.1)
$\left(q_{3},e_{1},1\right)$	(0.4,0.3,0.2)	(0.2,0.05,0.1)	(0.09,0.01,0.1)	(0.3,0.05,0.2)	(0.2,0.05,0.06)	(0.6,0.3,0.1)
$\left(q_{3},e_{2},1\right)$	(0.5,0.09,0.2)	(0.6,0.05,0.1)	(0.04,0.02,0.4)	(0.5,0.05,0.1)	(0.3,0.02,0.03)	(0.5,0.3,0.1)
$\left(q_{3},e_{3},1\right)$	(0.7,0.1,0.02)	(0.3,0.04,0.01)	(0.08,0.1,0.1)	(0.4,0.09,0.2)	(0.2,0.5,0.09)	(0.07,0.3,0.1)
$\left(q_{4},e_{1},1\right)$	(0.6,0.3,0.1)	(0.09,0.3,0.1)	(0.04,0.7,0.1)	(0.6,0.04,0.02)	(0.5,0.03,0.04)	(0.1,0.04,0.3)
$\left(q_{4},e_{2},1\right)$	(0.05,0.02,0.3)	(0.03,0.5,0.1)	(0.3,0.2,0.04)	(0.05,0.02,0.1)	(0.4,0.06,0.01)	(0.4,0.03,0.2)
$\left(q_{4},e_{3},1\right)$	(0.08,0.04,0.1)	(0.06,0.4,0.3)	(0.2,0.03,0.01)	(0.4,0.07,0.2)	(0.09,0.2,0.1)	(0.3,0.2,0.1)
$\left(q_{1},e_{1},0\right)$	(0.05,0.09,0.3)	(0.08,0.06,0.2)	(0.09,0.2,0.3)	(0.05,0.5,0.01)	(0.04,0.1,0.2)	(0.5,0.1,0.1)
$\left(q_{1},e_{2},0\right)$	(0.06,0.5,0.2)	(0.08,0.4,0.2)	(0.05,0.4,0.2)	(0.4,0.3,0.2)	(0.05,0.4,0.1)	(0.2,0.3,0.2)
$\left(q_{1},e_{3},0\right)$	(0.08,0.07,0.2)	(0.09,0.04,0.4)	(0.07,0.08,0.3)	(0.05,0.07,0.6)	(0.04,0.07,0.3)	(0.03,0.4,0.2)
$\left(q_{2},e_{1},0\right)$	(0.6,0.2,0.1)	(0.5,0.3,0.2)	(0.09,0.3,0.3)	(0.08,0.4,0.3)	(0.07,0.6,0.3)	(0.07,0.6,0.3)
$\left(q_{2},e_{2},0\right)$	(0.5,0.08,0.2)	(0.09,0.5,0.3)	(0.08,0.6,0.4)	(0.5,0.06,0.3)	(0.08,0.7,0.1)	(0.03,0.2,0.4)
$\left(q_{2},e_{3},0\right)$	(0.5,0.08,0.2)	(0.09,0.5,0.3)	(0.08,0.6,0.3)	(0.5,0.06,0.3)	(0.8,0.07,0.1)	(0.4,0.01,0.3)
$\left(q_{3},e_{1},0\right)$	(0.05,0.07,0.1)	(0.06,0.08,0.2)	(0.08,0.4,0.4)	(0.009,0.1,0.2)	(0.7,0.06,0.1)	(0.3,0.04,0.1)
$\left(q_{3},e_{2},0\right)$	(0.08,0.3,0.2)	(0.05,0.4,0.2)	(0.06,0.08,0.2)	(0.05,0.06,0.4)	(0.09,0.3,0.5)	(0.03,0.3,0.2)
$\left(q_{3},e_{3},0\right)$	(0.3,0.1,0.4)	(0.06,0.6,0.4)	(0.3,0.4,0.1)	(0.08,0.4,0.2)	(0.2,0.4,0.2)	(0.2,0.4,0.3)
$\left(q_{4},e_{1},0\right)$	(0.05,0.4,0.5)	(0.08,0.05,0.1)	(0.07,0.2,0.3)	(0.5,0.06,0.4)	(0.4,0.3,0.1)	(0.2,0.3,0.3)
$\left(q_{4},e_{2},0\right)$	(0.08,0.04,0.3)	(0.3,0.03,0.2)	(0.6,0.2,0.1)	(0.7,0.1,0.1)	(0.5,0.2,0.2)	(0.4,0.3,0.2)
$\left(q_{4},e_{3},0\right)$	(0.4,0.5,0.1)	(0.4,0.3,0.3)	(0.5,0.2,0.1)	(0.5,0.1,0.3)	(0.2,0.1,0.4)	(0.2,0.4,0.3)

**Table 11:** Tabular form of the PFSES (Y,A).

(Y,A)	$x_{1}$	$x_{2}$	$x_{3}$	$x_{4}$	$x_{5}$	$x_{6}$
$\left(q_{1},e_{3},1\right)$	(0.7,0.1,0.1)	(0.4,0.2,0.2)	(0.5,0.1,0.3)	(0.3,0.3,0.2)	(0.7,0.1,0.2)	(0.1,0.5,0.3)
$\left(q_{2},e_{1},1\right)$	(0.2,0.2,0.3)	(0.2,0.1,0.5)	(0.2,0.3,0.4)	(0.3,0.1,0.2)	(0.5,0.3,0.1)	(0.5,0.2,0.3)
$\left(q_{3},e_{2},0\right)$	(0.5,0.09,0.2)	(0.6,0.05,0.1)	(0.04,0.02,0.4)	(0.5,0.05,0.1)	(0.3,0.02,0.03)	(0.5,0.3,0.1)
$\left(q_{4},e_{1},0\right)$	(0.05,0.4,0.5)	(0.08,0.05,0.1)	(0.07,0.2,0.3)	(0.5,0.06,0.4)	(0.4,0.3,0.1)	(0.2,0.3,0.3)

**Table 12:** Tabular arrangement of the PFSES (Ω,B).

(Ω,B)	$x_{1}$	$x_{2}$	$x_{3}$	$x_{4}$	$x_{5}$	$x_{6}$
$\left(q_{1},e_{2},1\right)$	(0.6,0.3,0.1)	(0.7,0.2,0.1)	(0.2,0.6,0.1)	(0.6,0.3,0.01)	(0.4,0.5,0.02)	(0.4,0.3,0.05)
$\left(q_{2},e_{2},1\right)$	(0.4,0.4,0.1)	(0.3,0.6,0.01)	(0.5,0.3,0.02)	(0.5,0.4,0.02)	(0.4,0.5,0.03)	(0.3,0.6,0.05)
$\left(q_{3},e_{1},0\right)$	(0.2,0.3,0.04)	(0.2,0.4,0.1)	(0.2,0.5,0.05)	(0.3,0.4,0.1)	(0.8,0.1,0.04)	(0.5,0.2,0.09)
$\left(q_{4},e_{3},0\right)$	(0.4,0.5,0.1)	(0.5,0.4,0.1)	(0.6,0.3,0.04)	(0.6,0.2,0.2)	(0.4,0.4,0.09)	(0.3,0.5,0.08)

**Table 13:** The operation ‘AND’ between PFSESs (Y,A) and (Ω,B).

$\left(Y,A\right)\bar{\wedge }\left(\Omega ,B\right)$	$x_{1}$	$x_{2}$	$x_{3}$	$x_{4}$	$x_{5}$	$x_{6}$
$\left(\left(q_{1},e_{3},1\right),\left(q_{1},e_{2},1\right)\right)$	(0.6,0.1,0.1)	(0.4,0.2,0.2)	(0.2,0.1,0.3)	(0.3,0.3,0.2)	(0.4,0.1,0.2)	(0.1,0.3,0.3)
$\left(\left(q_{1},e_{3},1\right),\left(q_{2},e_{2},1\right)\right)$	(0.4,0.1,0.1)	(0.3,0.2,0.2)	(0.5,0.1,0.3)	(0.3,0.3,0.2)	(0.4,0.1,0.2)	(0.1,0.5,0.3)
$\left(\left(q_{1},e_{3},1\right),\left(q_{3},e_{1},0\right)\right)$	(0.2,0.1,0.1)	(0.2,0.2,0.2)	(0.2,0.1,0.3)	(0.3,0.3,0.2)	(0.7,0.1,0.2)	(0.1,0.2,0.3)
$\left(\left(q_{1},e_{3},1\right),\left(q_{4},e_{3},0\right)\right)$	(0.4,0.1,0.1)	(0.4,0.2,0.2)	(0.5,0.1,0.3)	(0.3,0.2,0.2)	(0.4,0.1,0.2)	(0.1,0.5,0.3)
$\left(\left(q_{2},e_{1},1\right),\left(q_{1},e_{2},1\right)\right)$	(0.2,0.2,0.3)	(0.2,0.1,0.5)	(0.2,0.3,0.4)	(0.3,0.1,0.2)	(0.4,0.3,0.1)	(0.4,0.2,0.3)
$\left(\left(q_{2},e_{1},1\right),\left(q_{2},e_{2},1\right)\right)$	(0.2,0.2,0.3)	(0.2,0.1,0.5)	(0.2,0.3,0.4)	(0.3,0.1,0.2)	(0.4,0.3,0.1)	(0.3,0.2,0.3)
$\left(\left(q_{2},e_{1},1\right),\left(q_{3},e_{1},0\right)\right)$	(0.2,0.2,0.3)	(0.2,0.1,0.5)	(0.2,0.3,0.4)	(0.3,0.1,0.2)	(0.5,0.1,0.1)	(0.5,0.2,0.3)
$\left(\left(q_{2},e_{1},1\right),\left(q_{4},e_{3},0\right)\right)$	(0.2,0.2,0.3)	(0.2,0.1,0.5)	(0.2,0.3,0.4)	(0.3,0.1,0.2)	(0.4,0.3,0.1)	(0.3,0.2,0.3)
$\left(\left(q_{3},e_{2},0\right),\left(q_{1},e_{2},1\right)\right)$	(0.5,0.09,0.2)	(0.6,0.05,0.1)	(0.04,0.02,0.4)	(0.5,0.05,0.1)	(0.3,0.02,0.03)	(0.4,0.3,0.1)
$\left(\left(q_{3},e_{2},0\right),\left(q_{2},e_{2},1\right)\right)$	(0.4,0.09,0.2)	(0.3,0.05,0.1)	(0.04,0.02,0.4)	(0.5,0.05,0.1)	(0.3,0.02,0.03)	(0.3,0.3,0.1)
$\left(\left(q_{3},e_{2},0\right),\left(q_{3},e_{1},0\right)\right)$	(0.2,0.09,0.2)	(0.2,0.05,0.1)	(0.04,0.02,0.4)	(0.3,0.05,0.1)	(0.3,0.02,0.04)	(0.5,0.2,0.1)
$\left(\left(q_{3},e_{2},0\right),\left(q_{4},e_{3},0\right)\right)$	(0.4,0.09,0.2)	(0.5,0.05,0.1)	(0.04,0.02,0.4)	(0.5,0.05,0.2)	(0.3,0.02,0.09)	(0.3,0.3,0.1)
$\left(\left(q_{4},e_{1},0\right),\left(q_{1},e_{2},1\right)\right)$	(0.05,0.3,0.5)	(0.08,0.05,0.1)	(0.07,0.2,0.3)	(0.5,0.06,0.4)	(0.4,0.3,0.1)	(0.2,0.3,0.3)
$\left(\left(q_{4},e_{1},0\right),\left(q_{2},e_{2},1\right)\right)$	(0.05,0.4,0.5)	(0.08,0.05,0.1)	(0.07,0.2,0.3)	(0.5,0.06,0.4)	(0.4,0.3,0.1)	(0.2,0.3,0.3)
$\left(\left(q_{4},e_{1},0\right),\left(q_{3},e_{1},0\right)\right)$	(0.05,0.3,0.5)	(0.08,0.05,0.1)	(0.07,0.2,0.3)	(0.3,0.06,0.4)	(0.4,0.1,0.1)	(0.2,0.2,0.3)
$\left(\left(q_{4},e_{1},0\right),\left(q_{4},e_{3},0\right)\right)$	(0.05,0.4,0.5)	(0.08,0.05,0.1)	(0.07,0.2,0.3)	(0.5,0.06,0.4)	(0.4,0.3,0.1)	(0.2,0.3,0.3)

**Table 14:** The operation ‘OR’ between PFSESs (Y,A) and (Ω,B).

$\left(Y,A\right)\underline{\vee }\left(\Omega ,B\right)$	$x_{1}$	$x_{2}$	$x_{3}$	$x_{4}$	$x_{5}$	$x_{6}$
$\left(\left(q_{1},e_{3},1\right),\left(q_{1},e_{2},1\right)\right)$	(0.7,0.1,0.1)	(0.7,0.2,0.1)	(0.5,0.1,0.1)	(0.6,0.3,0.01)	(0.7,0.1,0.02)	(0.4,0.3,0.05)
$\left(\left(q_{1},e_{3},1\right),\left(q_{2},e_{2},1\right)\right)$	(0.7,0.1,0.1)	(0.4,0.2,0.01)	(0.5,0.1,0.02)	(0.5,0.3,0.02)	(0.7,0.1,0.03)	(0.3,0.5,0.05)
$\left(\left(q_{1},e_{3},1\right),\left(q_{3},e_{1},0\right)\right)$	(0.7,0.1,0.04)	(0.4,0.2,0.1)	(0.5,0.1,0.05)	(0.3,0.3,0.1)	(0.8,0.1,0.04)	(0.5,0.2,0.09)
$\left(\left(q_{1},e_{3},1\right),\left(q_{4},e_{3},0\right)\right)$	(0.7,0.1,0.1)	(0.5,0.2,0.1)	(0.6,0.1,0.04)	(0.6,0.2,0.2)	(0.7,0.1,0.09)	(0.3,0.5,0.08)
$\left(\left(q_{2},e_{1},1\right),\left(q_{1},e_{2},1\right)\right)$	(0.6,0.2,0.1)	(0.7,0.1,0.1)	(0.2,0.3,0.1)	(0.6,0.1,0.01)	(0.5,0.3,0.02)	(0.5,0.2,0.05)
$\left(\left(q_{2},e_{1},1\right),\left(q_{2},e_{2},1\right)\right)$	(0.4,0.2,0.1)	(0.3,0.1,0.01)	(0.5,0.3,0.02)	(0.5,0.1,0.02)	(0.5,0.3,0.03)	(0.5,0.2,0.05)
$\left(\left(q_{2},e_{1},1\right),\left(q_{3},e_{1},0\right)\right)$	(0.2,0.2,0.04)	(0.2,0.1,0.1)	(0.2,0.3,0.05)	(0.3,0.1,0.1)	(0.8,0.1,0.04)	(0.5,0.2,0.09)
$\left(\left(q_{2},e_{1},1\right),\left(q_{4},e_{3},0\right)\right)$	(0.4,0.2,0.1)	(0.5,0.1,0.1)	(0.6,0.3,0.04)	(0.6,0.1,0.2)	(0.5,0.3,0.09)	(0.5,0.2,0.08)
$\left(\left(q_{3},e_{2},0\right),\left(q_{1},e_{2},1\right)\right)$	(0.6,0.09,0.1)	(0.7,0.05,0.1)	(0.2,0.02,0.1)	(0.6,0.05,0.01)	(0.4,0.02,0.02)	(0.5,0.3,0.05)
$\left(\left(q_{3},e_{2},0\right),\left(q_{2},e_{2},1\right)\right)$	(0.5,0.09,0.1)	(0.6,0.05,0.01)	(0.5,0.02,0.02)	(0.5,0.05,0.02)	(0.4,0.02,0.03)	(0.5,0.3,0.05)
$\left(\left(q_{3},e_{2},0\right),\left(q_{3},e_{1},0\right)\right)$	(0.5,0.09,0.04)	(0.6,0.05,0.1)	(0.2,0.02,0.05)	(0.5,0.05,0.1)	(0.8,0.02,0.03)	(0.5,0.2,0.09)
$\left(\left(q_{3},e_{2},0\right),\left(q_{4},e_{3},0\right)\right)$	(0.5,0.09,0.1)	(0.6,0.05,0.1)	(0.6,0.02,0.04)	(0.6,0.05,0.1)	(0.4,0.02,0.03)	(0.5,0.3,0.08)
$\left(\left(q_{4},e_{1},0\right),\left(q_{1},e_{2},1\right)\right)$	(0.6,0.3,0.1)	(0.7,0.05,0.1)	(0.2,0.2,0.1)	(0.6,0.06,0.01)	(0.4,0.3,0.02)	(0.4,0.3,0.05)
$\left(\left(q_{4},e_{1},0\right),\left(q_{2},e_{2},1\right)\right)$	(0.4,0.4,0.1)	(0.3,0.05,0.01)	(0.5,0.2,0.02)	(0.5,0.06,0.02)	(0.4,0.3,0.03)	(0.3,0.3,0.05)
$\left(\left(q_{4},e_{1},0\right),\left(q_{3},e_{1},0\right)\right)$	(0.2,0.3,0.04)	(0.2,0.05,0.1)	(0.2,0.2,0.05)	(0.5,0.06,0.1)	(0.8,0.1,0.04)	(0.5,0.2,0.09)
$\left(\left(q_{4},e_{1},0\right),\left(q_{4},e_{3},0\right)\right)$	(0.4,0.4,0.1)	(0.5,0.05,0.1)	(0.6,0.2,0.04)	(0.6,0.06,0.2)	(0.4,0.3,0.09)	(0.3,0.3,0.08)

**Table 15:** Tabular representation of an agree-PFSES ${\left(Y,A\right)}_{1}$.

${\left(Y,A\right)}_{1}$	$x_{1}$	$x_{2}$	$x_{3}$	$x_{4}$	$x_{5}$	$x_{6}$
$\left(q_{1},e_{1},1\right)$	(0.2,0.3,0.4)	(0.1,0.5,0.04)	(0.3,0.1,0.07)	(0.2,0.1,0.3)	(0.3,0.3,0.4)	(0.4,0.2,0.1)
$\left(q_{1},e_{2},1\right)$	(0.2,0.1,0.5)	(0.2,0.2,0.3)	(0.5,0.2,0.3)	(0.3,0.1,0.2)	(0.1,0.5,0.3)	(0.2,0.4,0.1)
$\left(q_{1},e_{3},1\right)$	(0.4,0.4,0.2)	(0.7,0.1,0.1)	(0.5,0.3,0.1)	(0.7,0.1,0.2)	(0.3,0.3,0.2)	(0.3,0.2,0.1)
$\left(q_{2},e_{1},1\right)$	(0.4,0.2,0.2)	(0.1,0.2,0.5)	(0.3,0.5,0.1)	(0.4,0.3,0.2)	(0.6,0.2,0.1)	(0.2,0.4,0.2)
$\left(q_{2},e_{2},1\right)$	(0.5,0.1,0.3)	(0.6,0.1,0.1)	(0.5,0.3,0.1)	(0.4,0.2,0.2)	(0.1,0.5,0.1)	(0.2,0.3,0.4)
$\left(q_{2},e_{3},1\right)$	(0.5,0.2,0.2)	(0.04,0.1,0.2)	(0.3,0.2,0.1)	(0.09,0.2,0.3)	(0.08,0.06,0.2)	(0.03,0.5,0.1)
$\left(q_{3},e_{1},1\right)$	(0.2,0.5,0.09)	(0.6,0.04,0.02)	(0.06,0.4,0.3)	(0.4,0.07,0.2)	(0.09,0.3,0.1)	(0.5,0.1,0.3)
$\left(q_{3},e_{2},1\right)$	(0.05,0.02,0.3)	(0.08,0.04,0.1)	(0.5,0.1,0.1)	(0.09,0.2,0.1)	(0.6,0.3,0.1)	(0.05,0.5,0.01)
$\left(q_{3},e_{3},1\right)$	(0.2,0.05,0.1)	(0.4,0.06,0.01)	(0.5,0.3,0.1)	(0.5,0.03,0.04)	(0.05,0.02,0.1)	(0.3,0.04,0.01)
$\left(q_{4},e_{1},1\right)$	(0.08,0.1,0.1)	(0.1,0.04,0.3)	(0.05,0.09,0.3)	(0.7,0.1,0.02)	(0.2,0.03,0.01)	(0.4,0.03,0.2)
$\left(q_{4},e_{2},1\right)$	(0.5,0.09,0.2)	(0.07,0.3,0.1)	(0.04,0.02,0.4)	(0.3,0.02,0.03)	(0.5,0.05,0.1)	(0.3,0.2,0.04)
$\left(q_{4},e_{3},1\right)$	(0.6,0.05,0.1)	(0.4,0.3,0.2)	(0.04,0.7,0.1)	(0.4,0.09,0.2)	(0.09,0.01,0.1)	(0.07,0.3,0.1)
$\left(q_{5},e_{1},1\right)$	(0.08,0.04,0.3)	(0.05,0.4,0.5)	(0.08,0.3,0.2)	(0.2,0.1,0.4)	(0.3,0.05,0.2)	(0.07,0.2,0.3)
$\left(q_{5},e_{2},1\right)$	(0.2,0.05,0.06)	(0.06,0.6,0.4)	(0.2,0.4,0.3)	(0.6,0.2,0.1)	(0.3,0.03,0.2)	(0.7,0.1,0.1)
$\left(q_{5},e_{3},1\right)$	(0.6,0.3,0.1)	(0.009,0.1,0.2)	(0.06,0.08,0.2)	(0.08,0.4,0.2)	(0.4,0.3,0.2)	(0.5,0.2,0.2)
$\left(q_{6},e_{1},1\right)$	(0.05,0.4,0.2)	(0.2,0.3,0.3)	(0.4,0.3,0.1)	(0.5,0.06,0.4)	(0.3,0.1,0.4)	(0.3,0.4,0.1)
$\left(q_{6},e_{2},1\right)$	(0.2,0.4,0.2)	(0.4,0.5,0.1)	(0.5,0.2,0.1)	(0.03,0.3,0.2)	(0.4,0.3,0.3)	(0.05,0.06,0.4)
$\left(q_{6},e_{3},1\right)$	(0.7,0.06,0.1)	(0.09,0.3,0.5)	(0.5,0.1,0.3)	(0.08,0.05,0.1)	(0.2,0.4,0.3)	(0.3,0.04,0.1)
$\left(q_{7},e_{1},1\right)$	(0.3,0.04,0.07)	(0.4,0.09,0.09)	(0.2,0.4,0.06)	(0.5,0.03,0.01)	(0.3,0.2,0.05)	(0.2,0.5,0.03)
$\left(q_{7},e_{2},1\right)$	(0.2,0.07,0.05)	(0.4,0.04,0.07)	(0.2,0.4,0.06)	(0.5,0.06,0.04)	(0.2,0.5,0.01)	(0.4,0.08,0.04)
$\left(q_{7},e_{3},1\right)$	(0.4,0.4,0.06)	(0.1,0.09,0.06)	(0.2,0.1,0.04)	(0.3,0.07,0.1)	(0.3,0.3,0.06)	(0.5,0.3,0.03)
$\left(q_{8},e_{1},1\right)$	(0.6,0.1,0.04)	(0.1,0.7,0.06)	(0.7,0.1,0.01)	(0.4,0.1,0.04)	(0.7,0.2,0.05)	(0.3,0.07,0.08)
$\left(q_{8},e_{2},1\right)$	(0.2,0.3,0.05)	(0.3,0.04,0.01)	(0.1,0.02,0.06)	(0.6,0.3,0.06)	(0.3,0.2,0.01)	(0.6,0.3,0.06)
$\left(q_{8},e_{3},1\right)$	(0.3,0.5,0.01)	(0.7,0.03,0.01)	(0.5,0.07,0.01)	(0.7,0.04,0.01)	(0.3,0.02,0.06)	(0.5,0.06,0.01)
$\left(q_{9},e_{1},1\right)$	(0.5,0.3,0.03)	(0.3,0.1,0.05)	(0.6,0.09,0.03)	(0.2,0.01,0.09)	(0.1,0.02,0.09)	(0.5,0.05,0.03)
$\left(q_{9},e_{2},1\right)$	(0.4,0.05,0.05)	(0.7,0.05,0.02)	(0.5,0.09,0.07)	(0.6,0.3,0.01)	(0.6,0.05,0.02)	(0.5,0.3,0.01)
$\left(q_{9},e_{3},1\right)$	(0.7,0.2,0.03)	(0.4,0.3,0.03)	(0.7,0.1,0.02)	(0.4,0.02,0.01)	(0.4,0.05,0.02)	(0.3,0.3,0.08)
$\left(q_{10},e_{1},1\right)$	(0.2,0.3,0.03)	(0.4,0.3,0.1)	(0.5,0.4,0.02)	(0.7,0.2,0.04)	(0.3,0.4,0.01)	(0.4,0.04,0.01)
$\left(q_{10},e_{2},1\right)$	(0.5,0.2,0.02)	(0.2,0.4,0.1)	(0.6,0.1,0.02)	(0.6,0.3,0.01)	(0.4,0.3,0.05)	(0.7,0.2,0.03)
$\left(q_{10},e_{3},1\right)$	(0.2,0.4,0.01)	(0.2,0.5,0.05)	(0.2,0.3,0.04)	(0.4,0.2,0.08)	(0.2,0.7,0.05)	(0.4,0.2,0.02)
$\left(q_{11},e_{1},1\right)$	(0.4,0.2,0.05)	(0.2,0.5,0.05)	(0.7,0.1,0.02)	(0.5,0.06,0.04)	(0.2,0.4,0.05)	(0.3,0.4,0.05)
$\left(q_{11},e_{2},1\right)$	(0.3,0.5,0.01)	(0.4,0.05,0.02)	(0.2,0.5,0.06)	(0.6,0.1,0.09)	(0.4,0.1,0.09)	(0.4,0.5,0.06)
$\left(q_{11},e_{3},1\right)$	(0.2,0.7,0.04)	(0.7,0.2,0.03)	(0.6,0.1,0.02)	(0.3,0.6,0.01)	(0.9,0.08,0.02)	(0.1,0.7,0.06)
$\left(q_{12},e_{1},1\right)$	(0.4,0.2,0.07)	(0.3,0.4,0.1)	(0.2,0.7,0.03)	(0.5,0.2,0.04)	(0.4,0.5,0.08)	(0.2,0.3,0.04)
$\left(q_{12},e_{2},1\right)$	(0.3,0.5,0.03)	(0.5,0.2,0.2)	(0.3,0.5,0.09)	(0.8,0.1,0.04)	(0.3,0.5,0.09)	(0.2,0.7,0.06)
$\left(q_{12},e_{3},1\right)$	(0.2,0.3,0.05)	(0.2,0.6,0.01)	(0.2,0.5,0.06)	(0.3,0.4,0.06)	(0.5,0.2,0.01)	(0.4,0.3,0.04)

**Table 16:** Tabular representation of a disagree-PFSES ${\left(Y,A\right)}_{0}$.

${\left(Y,A\right)}_{0}$	$x_{1}$	$x_{2}$	$x_{3}$	$x_{4}$	$x_{5}$	$x_{6}$
$\left(q_{1},e_{1},0\right)$	(0.1,0.3,0.3)	(0.2,0.03,0.4)	(0.3,0.04,0.1)	(0.1,0.05,0.6)	(0.1,0.05,0.2)	(0.1,0.01,0.09)
$\left(q_{1},e_{2},0\right)$	(0.1,0.3,0.5)	(0.2,0.05,0.3)	(0.1,0.1,0.081)	(0.4,0.3,0.2)	(0.09,0.5,0.2)	(0.2,0.09,0.5)
$\left(q_{1},e_{3},0\right)$	(0.1,0.1,0.7)	(0.3,0.1,0.5)	(0.4,0.3,0.2)	(0.2,0.1,0.7)	(0.2,0.3,0.3)	(0.3,0.2,0.2)
$\left(q_{2},e_{1},0\right)$	(0.2,0.2,0.4)	(0.2,0.1,0.3)	(0.1,0.4,0.2)	(0.3,0.5,0.1)	(0.1,0.4,0.2)	(0.5,0.1,0.2)
$\left(q_{2},e_{2},0\right)$	(0.4,0.3,0.3)	(0.1,0.5,0.1)	(0.2,0.2,0.4)	(0.1,0.2,0.4)	(0.4,0.3,0.2)	(0.2,0.5,0.2)
$\left(q_{2},e_{3},0\right)$	(0.1,0.2,0.3)	(0.3,0.2,0.5)	(0.2,0.4,0.4)	(0.1,0.1,0.6)	(0.1,0.3,0.5)	(0.1,0.1,0.6)
$\left(q_{3},e_{1},0\right)$	(0.1,0.2,0.6)	(0.1,0.3,0.5)	(0.1,0.5,0.3)	(0.2,0.2,0.5)	(0.2,0.4,0.2)	(0.5,0.2,0.1)
$\left(q_{3},e_{2},0\right)$	(0.3,0.1,0.2)	(0.2,0.2,0.6)	(0.03,0.02,0.3)	(0.1,0.05,0.5)	(0.1,0.1,0.6)	(0.4,0.02,0.04)
$\left(q_{3},e_{3},0\right)$	(0.1,0.1,0.6)	(0.3,0.02,0.05)	(0.02,0.04,0.6)	(0.1,0.3,0.6)	(0.2,0.03,0.4)	(0.2,0.3,0.4)
$\left(q_{4},e_{1},0\right)$	(0.01,0.03,0.2)	(0.1,0.02,0.05)	(0.3,0.4,0.06)	(0.2,0.07,0.4)	(0.1,0.07,0.8)	(0.4,0.1,0.3)
$\left(q_{4},e_{2},0\right)$	(0.1,0.2,0.6)	(0.2,0.3,0.08)	(0.1,0.06,0.7)	(0.1,0.4,0.3)	(0.3,0.5,0.09)	(0.1,0.04,0.3)
$\left(q_{4},e_{3},0\right)$	(0.5,0.4,0.05)	(0.2,0.08,0.5)	(0.2,0.3,0.03)	(0.5,0.3,0.09)	(0.2,0.08,0.06)	(0.4,0.4,0.08)
$\left(q_{5},e_{1},0\right)$	(0.1,0.07,0.05)	(0.2,0.1,0.009)	(0.4,0.6,0.06)	(0.2,0.3,0.4)	(0.2,0.4,0.05)	(0.3,0.6,0.08)
$\left(q_{5},e_{2},0\right)$	(0.2,0.3,0.5)	(0.4,0.06,0.05)	(0.3,0.5,0.09)	(0.1,0.2,0.6)	(0.1,0.05,0.08)	(0.2,0.4,0.08)
$\left(q_{5},e_{3},0\right)$	(0.3,0.04,0.08)	(0.1,0.5,0.4)	(0.1,0.2,0.5)	(0.3,0.1,0.5)	(0.2,0.08,0.5)	(0.2,0.4,0.2)
$\left(q_{6},e_{1},0\right)$	(0.4,0.5,0.1)	(0.7,0.2,0.1)	(0.7,0.2,0.1)	(0.4,0.5,0.1)	(0.4,0.4,0.1)	(0.2,0.6,0.1)
$\left(q_{6},e_{2},0\right)$	(0.2,0.07,0.08)	(0.4,0.06,0.5)	(0.3,0.06,0.5)	(0.3,0.4,0.2)	(0.2,0.03,0.3)	(0.4,0.6,0.08)
$\left(q_{6},e_{3},0\right)$	(0.3,0.4,0.2)	(0.3,0.2,0.07)	(0.4,0.04,0.09)	(0.1,0.7,0.08)	(0.6,0.07,0.05)	(0.3,0.08,0.07)
$\left(q_{7},e_{1},0\right)$	(0.1,0.3,0.4)	(0.3,0.3,0.2)	(0.3,0.3,0.09)	(0.1,0.1,0.7)	(0.3,0.07,0.04)	(0.3,0.1,0.01)
$\left(q_{7},e_{2},0\right)$	(0.2,0.03,0.3)	(0.3,0.6,0.07)	(0.4,0.01,0.1)	(0.3,0.4,0.08)	(0.4,0.2,0.03)	(0.1,0.2,0.03)
$\left(q_{7},e_{3},0\right)$	(0.2,0.2,0.5)	(0.3,0.06,0.5)	(0.4,0.1,0.2)	(0.3,0.01,0.4)	(0.8,0.1,0.01)	(0.4,0.3,0.05)
$\left(q_{8},e_{1},0\right)$	(0.2,0.3,0.04)	(0.6,0.1,0.09)	(0.2,0.5,0.05)	(0.1,0.8,0.04)	(0.2,0.4,0.1)	(0.2,0.7,0.04)
$\left(q_{8},e_{2},0\right)$	(0.7,0.2,0.05)	(0.2,0.3,0.05)	(0.2,0.3,0.04)	(0.6,0.1,0.05)	(0.2,0.4,0.01)	(0.3,0.4,0.1)
$\left(q_{8},e_{3},0\right)$	(0.4,0.5,0.06)	(0.6,0.3,0.06)	(0.2,0.5,0.05)	(0.7,0.1,0.08)	(0.2,0.7,0.03)	(0.2,0.5,0.06)
$\left(q_{9},e_{1},0\right)$	(0.6,0.2,0.04)	(0.4,0.2,0.07)	(0.3,0.5,0.08)	(0.9,0.08,0.02)	(0.6,0.1,0.02)	(0.1,0.4,0.08)
$\left(q_{9},e_{2},0\right)$	(0.4,0.3,0.04)	(0.4,0.1,0.09)	(0.1,0.8,0.06)	(0.3,0.3,0.06)	(0.3,0.6,0.01)	(0.5,0.06,0.04)
$\left(q_{9},e_{3},0\right)$	(0.6,0.3,0.1)	(0.4,0.5,0.02)	(0.4,0.2,0.05)	(0.5,0.3,0.02)	(0.3,0.5,0.04)	(0.4,0.3,0.1)
$\left(q_{10},e_{1},0\right)$	(0.5,0.3,0.1)	(0.6,0.2,0.02)	(0.4,0.5,0.1)	(0.8,0.1,0.03)	(0.3,0.6,0.03)	(0.6,0.3,0.01)
$\left(q_{10},e_{2},0\right)$	(0.6,0.3,0.01)	(0.5,0.3,0.1)	(0.7,0.2,0.03)	(0.3,0.6,0.01)	(0.5,0.3,0.02)	(0.5,0.4,0.02)
$\left(q_{10},e_{3},0\right)$	(0.4,0.5,0.03)	(0.3,0.1,0.08)	(0.7,0.2,0.04)	(0.3,0.6,0.05)	(0.4,0.1,0.05)	(0.3,0.4,0.08)
$\left(q_{11},e_{1},0\right)$	(0.2,0.4,0.09)	(0.2,0.5,0.03)	(0.4,0.1,0.01)	(0.7,0.1,0.02)	(0.4,0.1,0.02)	(0.5,0.4,0.03)
$\left(q_{11},e_{2},0\right)$	(0.6,0.3,0.06)	(0.7,0.2,0.01)	(0.4,0.2,0.04)	(0.5,0.1,0.07)	(0.3,0.6,0.01)	(0.2,0.5,0.03)
$\left(q_{11},e_{3},0\right)$	(0.6,0.3,0.01)	(0.1,0.12,0.09)	(0.6,0.1,0.03)	(0.7,0.2,0.02)	(0.4,0.1,0.01)	(0.7,0.1,0.01)
$\left(q_{12},e_{1},0\right)$	(0.6,0.1,0.02)	(0.2,0.4,0.05)	(0.5,0.1,0.03)	(0.7,0.08,0.01)	(0.3,0.6,0.01)	(0.3,0.2,0.01)
$\left(q_{12},e_{2},0\right)$	(0.1,0.3,0.06)	(0.5,0.2,0.01)	(0.3,0.4,0.06)	(0.3,0.2,0.07)	(0.5,0.2,0.04)	(0.3,0.4,0.05)
$\left(q_{12},e_{3},0\right)$	(0.4,0.5,0.06)	(0.5,0.2,0.01)	(0.4,0.3,0.05)	(0.2,0.6,0.01)	(0.3,0.4,0.01)	(0.5,0.4,0.03)

**Table 17:** Score values of the agree-PFSES ${\left(Y,A\right)}_{1}$.

${\left(Y,A\right)}_{1}$	$x_{1}$	$x_{2}$	$x_{3}$	$x_{4}$	$x_{5}$	$x_{6}$
$\left(q_{1},e_{1},1\right)$	−0.5	−0.44	0.13	−0.2	−0.4	0.1
$\left(q_{1},e_{2},1\right)$	−0.4	−0.3	0	0	−0.7	−0.3
$\left(q_{1},e_{3},1\right)$	−0.2	0.5	0.1	0.4	−0.2	0
$\left(q_{2},e_{1},1\right)$	0	−0.6	−0.3	−0.1	0.3	−0.4
$\left(q_{2},e_{2},1\right)$	0.1	0.4	0.1	0	−0.5	−0.5
$\left(q_{2},e_{3},1\right)$	0.1	0.1	0	−0.41	−0.18	−0.57
$\left(q_{3},e_{1},1\right)$	−0.39	0.54	−0.64	0.13	−0.31	0.1
$\left(q_{3},e_{2},1\right)$	−0.27	−0.06	0.3	−0.21	0.2	−0.46
$\left(q_{3},e_{3},1\right)$	0.05	0.33	0.1	0.43	−0.07	0.25
$\left(q_{4},e_{1},1\right)$	−0.12	−0.24	−0.34	0.58	0.16	0.17
$\left(q_{4},e_{2},1\right)$	0.21	−0.33	−0.38	0.25	0.35	0.06
$\left(q_{4},e_{3},1\right)$	0.45	−0.1	0.76	0.11	−0.02	−0.33
$\left(q_{5},e_{1},1\right)$	−0.26	−0.85	−0.42	−0.3	0.05	−0.43
$\left(q_{5},e_{2},1\right)$	0.09	−0.94	−0.5	0.3	0.07	0.5
$\left(q_{5},e_{3},1\right)$	0.2	−0.291	−0.22	−0.52	−0.1	0.1
$\left(q_{6},e_{1},1\right)$	−0.55	−0.4	0	0.04	−0.2	−0.2
$\left(q_{6},e_{2},1\right)$	−0.4	−0.2	0.2	−0.47	−0.2	−0.41
$\left(q_{6},e_{3},1\right)$	0.54	−0.71	0.1	−0.07	−0.5	0.16
$\left(q_{7},e_{1},1\right)$	0.19	0.22	−0.26	0.46	0.05	−0.33
$\left(q_{7},e_{2},1\right)$	0.08	0.29	−0.26	0.4	−0.31	0.28
$\left(q_{7},e_{3},1\right)$	−0.06	−0.05	0.06	0.13	−0.06	0.17
$\left(q_{8},e_{1},1\right)$	0.46	−0.66	0.5	0.26	0.45	0.15
$\left(q_{8},e_{2},1\right)$	−0.15	0.25	0.02	0.24	0.09	0.24
$\left(q_{8},e_{3},1\right)$	−0.21	0.66	0.42	0.65	0.22	0.43
$\left(q_{9},e_{1},1\right)$	0.17	0.15	0.48	0.1	−0.01	0.42
$\left(q_{9},e_{2},1\right)$	0.3	0.63	0.34	0.29	0.53	0.19
$\left(q_{9},e_{3},1\right)$	0.47	0.07	0.58	0.37	0.33	−0.08
$\left(q_{10},e_{1},1\right)$	−0.13	0	0.08	0.46	−0.11	0.35
$\left(q_{10},e_{2},1\right)$	0.28	−0.3	0.48	0.29	0.05	0.47
$\left(q_{10},e_{3},1\right)$	−0.21	−0.35	−0.14	0.12	−0.55	0.18
$\left(q_{11},e_{1},1\right)$	0.15	−0.35	0.58	0.4	−0.25	−0.15
$\left(q_{11},e_{2},1\right)$	−0.21	0.33	−0.36	0.41	0.21	−0.16
$\left(q_{11},e_{3},1\right)$	−0.54	0.47	0.48	−0.31	0.8	−0.66
$\left(q_{12},e_{1},1\right)$	0.13	−0.2	−0.53	0.26	−0.18	−0.14
$\left(q_{12},e_{2},1\right)$	−0.23	0.1	−0.29	0.66	−0.29	−0.56
$\left(q_{12},e_{3},1\right)$	−0.15	−0.5	−0.36	−0.16	0.29	0.06
$a_{j}=\sum_{i}e_{ij}$	$a_{1}=-1.01$	$a_{2}=-2.831$	$a_{3}=0.81$	$a_{4}=4.99$	$a_{5}=-0.99$	$a_{6}=-1.3$

**Table 18:** Score values of the disagree-PFSES ${\left(Y,A\right)}_{0}$.

${\left(Y,A\right)}_{0}$	$x_{1}$	$x_{2}$	$x_{3}$	$x_{4}$	$x_{5}$	$x_{6}$
$\left(q_{1},e_{1},0\right)$	−0.5	−0.23	0.16	−0.55	−0.15	0
$\left(q_{1},e_{2},0\right)$	−0.7	−0.15	−0.081	−0.1	−0.61	−0.39
$\left(q_{1},e_{3},0\right)$	−0.7	−0.3	−0.1	−0.6	−0.4	−0.1
$\left(q_{2},e_{1},0\right)$	−0.4	−0.2	−0.5	−0.3	−0.5	0.2
$\left(q_{2},e_{2},0\right)$	−0.2	−0.5	−0.4	−0.5	−0.1	−0.5
$\left(q_{2},e_{3},0\right)$	−0.4	−0.4	−0.6	−0.6	−0.7	−0.6
$\left(q_{3},e_{1},0\right)$	−0.7	−0.7	−0.7	−0.5	−0.4	0.2
$\left(q_{3},e_{2},0\right)$	0	−0.6	−0.29	−0.45	−0.6	0.34
$\left(q_{3},e_{3},0\right)$	−0.6	0.23	−0.62	−0.8	−0.23	−0.5
$\left(q_{4},e_{1},0\right)$	−0.22	0.03	−0.16	−0.27	−0.77	0
$\left(q_{4},e_{2},0\right)$	−0.7	−0.18	−0.66	−0.6	−0.29	−0.24
$\left(q_{4},e_{3},0\right)$	0.05	−0.38	−0.13	0.11	0.06	−0.08
$\left(q_{5},e_{1},0\right)$	−0.02	0.091	−0.26	−0.5	−0.25	−0.38
$\left(q_{5},e_{2},0\right)$	−0.6	0.29	−0.29	−0.7	−0.03	−0.28
$\left(q_{5},e_{3},0\right)$	0.18	−0.8	−0.6	−0.3	−0.38	−0.4
$\left(q_{6},e_{1},0\right)$	−0.2	0.4	0.4	−0.2	−0.1	−0.5
$\left(q_{6},e_{2},0\right)$	0.05	−0.16	−0.26	−0.3	−0.13	−0.28
$\left(q_{6},e_{3},0\right)$	−0.3	0.03	0.27	−0.68	0.48	0.15
$\left(q_{7},e_{1},0\right)$	−0.6	−0.2	−0.09	−0.7	0.19	0.19
$\left(q_{7},e_{2},0\right)$	−0.13	−0.37	0.29	−0.18	0.17	−0.13
$\left(q_{7},e_{3},0\right)$	−0.5	−0.26	0.1	−0.11	0.69	0.05
$\left(q_{8},e_{1},0\right)$	−0.14	0.41	−0.35	−0.74	−0.3	−0.54
$\left(q_{8},e_{2},0\right)$	0.45	−0.15	−0.14	0.45	−0.21	−0.2
$\left(q_{8},e_{3},0\right)$	−0.16	0.24	−0.35	0.52	−0.53	−0.36
$\left(q_{9},e_{1},0\right)$	0.36	0.13	−0.28	0.8	0.48	−0.38
$\left(q_{9},e_{2},0\right)$	0.06	0.21	−0.76	−0.06	−0.31	0.4
$\left(q_{9},e_{3},0\right)$	0.2	−0.12	0.15	0.18	−0.24	0
$\left(q_{10},e_{1},0\right)$	0.1	0.38	−0.2	0.67	−0.33	0.29
$\left(q_{10},e_{2},0\right)$	0.29	0.19	0.47	−0.31	0.18	0.08
$\left(q_{10},e_{3},0\right)$	−0.13	0.12	0.46	−0.35	0.25	−0.18
$\left(q_{11},e_{1},0\right)$	−0.29	−0.33	0.29	0.58	0.28	0.07
$\left(q_{11},e_{2},0\right)$	0.24	0.49	0.16	0.33	−0.31	−0.33
$\left(q_{11},e_{3},0\right)$	0.29	−0.11	0.47	0.48	0.29	0.59
$\left(q_{12},e_{1},0\right)$	0.48	−0.25	0.37	0.61	−0.31	0.09
$\left(q_{12},e_{2},0\right)$	−0.26	0.29	−0.16	0.03	0.26	−0.15
$\left(q_{12},e_{3},0\right)$	−0.16	0.29	0.05	−0.41	−0.11	0.07
$b_{j}=\sum_{i}e_{ij}$	$b_{1}=-5.86$	$b_{2}=-2.569$	$b_{3}=-4.341$	$b_{4}=-6.05$	$b_{5}=-4.96$	$b_{6}=-3.8$

**Table 19:** Final score table.

$a_{j}=\sum_{i}e_{\mathrm{ij}}$	$b_{j}=\sum_{i}e_{\mathrm{ij}}$	$z_{j}=a_{j}-b_{j}$
$a_{1}=-1.01$	$b_{1}=-5.86$	$z_{1}=4.85$
$a_{2}=-2.831$	$b_{2}=-2.569$	$z_{2}=-0.262$
$a_{3}=0.81$	$b_{3}=-4.341$	$z_{3}=5.151$
$a_{4}=4.99$	$b_{4}=-6.05$	$z_{4}=11.04$
$a_{5}=-0.99$	$b_{5}=-4.96$	$z_{5}=3.97$
$a_{6}=-1.3$	$b_{6}=-3.8$	$z_{6}=2.5$

**Table 20:** Comparison of existing models with proposed PFSES approach.

Models	$x_{1}$	$x_{2}$	$x_{3}$	$x_{4}$	$x_{5}$	$x_{6}$	Rankings	Best Alternative
IFSESs [38]	4.33	0.19	3.21	7.21	2.40	1.17	$x_{4}>x_{1}>x_{3}>x_{5}>x_{6}>x_{2}$	$x_{4}$
Proposed PFSESs	4.85	−0.26	5.15	11.0	3.97	2.50	$x_{4}>x_{3}>x_{1}>x_{5}>x_{6}>x_{2}$	$x_{4}$

**Table 21:** Comparison with existing hybrid models.

Models	t = Number of Experts	Type of Data
Soft sets [25]	t=1	Discrete data in soft form
SESs [33]	t≥1	Discrete data in SES environment
FSESs [34]	t≥1	Data in FSES form
IFSESs [38]	t≥1	Data in IFSES environment
Proposed PFSESs	t≥1	Data in PFSES form

## Data Availability

The data used to support the findings of this study are included within the article.
